# Brown Adipocyte Sheets Alleviate Myocardial Ischemia–Reperfusion Injury Through NRG4–ErbB4–Dependent Ferroptosis Inhibition

**DOI:** 10.1002/advs.75588

**Published:** 2026-05-28

**Authors:** Lifu Sun, Yuting Liu, Jingbo Zhang, Takuji Kawamura, Junjun Li, Li Liu, Shigeru Miyagawa

**Affiliations:** ^1^ Department of Cardiovascular Surgery Graduate School of Medicine The University of Osaka Suita Osaka Japan; ^2^ Photonics Cell Evaluation Laboratory Graduate School of Engineering The University of Osaka Suita Osaka Japan

**Keywords:** brown adipocyte sheets, ferroptosis, myocardial ischemia–reperfusion injury, NRG4–ErbB4

## Abstract

Myocardial ischemia–reperfusion (I/R) injury causes cardiomyocyte death and cardiac dysfunction in part through ferroptosis. Brown adipocytes (BAs) have emerged as endocrine regulators with cardioprotective potential, yet their involvement in ferroptosis modulation during I/R injury remains unclear. Here, we engineered BA sheets and transplanted them onto the ischemic myocardium in a rat I/R model to evaluate therapeutic efficacy. BA sheets transplantation significantly improved cardiac function, reduced infarct size and fibrosis, and mitigated adverse remodeling while enhancing angiogenesis. In vitro, conditioned medium derived from BA sheets promoted cardiomyocyte survival, preserved contractile performance, and inhibited apoptosis and ferroptosis under hypoxia/reoxygenation stress. Mechanistically, these effects were mediated by the activation of the NRG4–ErbB4 axis and its downstream PI3K/AKT and NRF2/HO‐1 antioxidant signaling pathways. Our findings demonstrate that engineered BA sheets exert potent cardioprotection against myocardial I/R injury by suppressing ferroptosis in an NRG4–ErbB4–dependent manner, supporting their promise as a therapeutic strategy for ischemic heart disease.

## Graphical abstract

Brown adipocyte sheets are engineered to protect the heart against myocardial ischemia–reperfusion injury by restraining ferroptosis. Upon transplantation onto the cardiac surface, they improve cardiac function, limit infarction and fibrosis, and enhance angiogenesis. These effects are mediated by the NRG4–ErbB4 axis, which activates PI3K/AKT and NRF2/HO‐1 pathways, revealing a ferroptosis–targeting bioengineered strategy for cardiac repair.

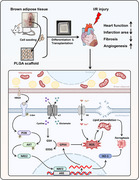

## 1 Introduction

Ischemic heart disease is the leading cause of morbidity and mortality worldwide, with myocardial infarction (MI) affecting approximately 15 million people annually [1]. MI leads to irreversible loss of cardiomyocytes and significantly impairs heart function [2]. Although reperfusion strategies such as thrombolysis and percutaneous coronary intervention (PCI) are essential to salvage the ischemic myocardium, they can paradoxically exacerbate myocardial injury, termed myocardial ischemia–reperfusion (I/R) injury [3]. Myocardial I/R injury may account for more than half of the final infarct size and promote adverse ventricular remodeling, underscoring the need for effective myocardial protection [4]. Various types of cell death occur during reperfusion, including necrosis, apoptosis, and autophagy [5, 6, 7]. However, ferroptosis is the predominant form of cell death during the medium‐to‐late phase of myocardial I/R injury, highlighting it as a critical therapeutic target [8]. Ferroptosis is characterized by iron‐dependent lipid peroxidation and is implicated in multiple organs and tissue types, including the brain, liver, kidney, and heart [9, 10, 11, 12]. Unlike other cell death mechanisms, ferroptosis has distinct molecular mechanisms and a pathophysiological significance: it is triggered by the accumulation of intracellular iron and the excessive production of reactive oxygen species (ROS), leading to lipid peroxidation of polyunsaturated fatty acids on cell membranes and organelles [9]. This process damages the membrane structure and results in cell death.

Brown adipose tissue (BAT) is a specialized adipose tissue that is regarded as an endocrine organ with distinctive structural and functional properties [13]. Unlike white adipose tissue (WAT), BAT is rich in mitochondria and expresses uncoupling protein‐1 (UCP‐1), which enables it to generate heat and secrete various bioactive molecules [14]. These molecules, including adipokines, cytokines, and extracellular vesicles, can target distant tissues and organs, such as the white adipose tissue, liver, pancreas, skeletal muscle, and heart, and play a role in metabolic regulation [12, 13, 15, 16]. A recent study of more than 50000 clinical patients found that individuals with detectable BAT have significantly lower incidences of metabolic and cardiovascular diseases than those without detectable BAT [17]. Additionally, animal studies have discovered the beneficial effects of BAT on the heart through the release of factors such as 12,13‐diHOME, fibroblast growth factor‐21, bone morphogenetic protein 3b, and interleukin‐6 [18, 19, 20, 21]. Although BAT exerts cardioprotective effects, the role of BAT‐derived factors in protecting the myocardium from I/R injury and their relationship to ferroptosis have not yet been fully elucidated.

We, therefore, aimed to construct novel engineered brown adipocyte (BA) sheets and demonstrate that these sheets protect against myocardial I/R injury. We further confirm the inhibitory effect thereof on ferroptosis, and reveal that the protective effects are associated with the activation of the NRG4–ErbB4 axis and its downstream PI3K/AKT and NRF2/HO‐1 antioxidant signaling pathways, using both in vivo and in vitro models.

## 2 Materials and Methods

### 2.1 BAs Culture and Adipocyte Sheets Formation

BAT was obtained from the interscapular region, while WAT was collected from the inguinal region of 3‐day‐old rats (CLEA Japan, Inc., Shizuoka, Japan), and tissue processing and digestion were performed as previously described [22]. Brown or white preadipocytes were directly seeded onto poly(lactic‐co‐glycolic acid) (PLGA) fiber scaffolds, a biodegradable cell carrier suitable for animal transplantation [23], and induced to differentiate for 2 days using Dulbecco's Modified Eagle Medium (DMEM) with 10% fetal bovine serum (FBS), supplemented with 170 nM insulin, 1 nM triiodothyronine (T3), 0.5 mM IBMX, 1 µM dexamethasone, and 1 µM rosiglitazone. The medium was then replaced with maintenance medium (DMEM with 10% FBS supplemented with 170 nM insulin and 1 nM T3) and cells were cultured for an additional 3–4 days. BAs cultured in the dishes were subjected to the same differentiation induction and maintenance procedures.

### 2.2 Animal Model of Myocardial I/R Injury and Treatments

As previously described [23], an animal model of myocardial I/R injury was established using 8‐week‐old male wild‐type Sprague–Dawley rats (CLEA Japan Inc., Shizuoka, Japan). Briefly, the rats were fixed on a heating pad maintained at a constant temperature of 37°C. The rats were anesthetized by inhalation of 1.5% isoflurane and tracheal intubation with mechanical ventilation to maintain respiration. The precordial area was routinely disinfected with iodophor after removing the fur using scissors. Using sterile scissors, an incision was made at the site of the strongest heartbeat, and a thoracotomy was performed by blunt dissection between the fourth and fifth intercostal spaces to open the chest. The pericardium was opened, and a surgical slipknot was made on the left anterior descending (LAD) coronary artery using nonabsorbable 6‐0 polypropylene sutures (Ethicon, Johnson & Johnson) with a plastic rod placed within the knot to occlude the coronary artery, and the incision was then sutured.

After 45 min, the incision was reopened, the slip knot was untied, and the plastic rod was removed. The pericardium was sutured, excess air in the chest cavity was expelled, and the incision was sequentially closed. The rats were then returned to their rearing cages for continuous feeding. The model rats were randomly divided into five groups as follows: (1) thoracotomy without LAD ligation (Sham group), (2) I/R model without treatment (I/R group), (3) I/R model with BA sheets transplantation (I/R+BA group), (4) I/R model with brown preadipocyte sheets transplantation (I/R+PreBA), and (5) I/R model with white adipocyte sheets transplantation (I/R+WA). In the sheets transplantation groups, after removing the plastic rod, sheets were applied to the surface of the damaged myocardium, between the visceral and parietal pericardium. All the animal experiments were conducted in accordance with the guidelines for animal experimentation approved by the University of Osaka (No. 03‐092‐011).

### 2.3 Echocardiography

Echocardiographic imaging was performed as previously described [2]. Briefly, rats were anesthetized with 1.5% isoflurane via a face mask and placed on a heating pad. Transthoracic echocardiographic examinations were conducted by a blinded technician using a micro‐ultrasound system (Vivid i; GE Healthcare, WI). Following the guidelines of the American Society of Echocardiography, the left ventricular internal diameter at end‐systole (LVIDs) and the left ventricular internal diameter at end‐diastole (LVIDd) were measured from M‐mode images using the average of four consecutive cardiac cycles. The left ventricular end‐systolic volume (LVESV) and end‐diastolic volume (LVEDV) were calculated using the Teichholz method:

$$\mathrm{LVESV}\;=\frac{7\times \mathrm{LVID}s^{3}}{2.4+\mathrm{LVIDs}}\;\;\mathrm{LVEDV}\;=\frac{7\times \mathrm{LVID}d^{3}}{2.4+\mathrm{LVIDd}}$$

Other parameters, such as ejection fraction and fractional shortening, were also calculated:

$$\begin{array}{ccc} \mathrm{LVEF}\; & = & 100\times \frac{\mathrm{LVEDV}-\mathrm{LVESV}}{\mathrm{LVEDV}}\;\mathrm{LVFS}\;\\ & = & 100\times \frac{\mathrm{LVIDd}-\mathrm{LVIDs}}{\mathrm{LVIDd}} \end{array}$$

### 2.4 2,3,5‐triphenyltetrazolium Chloride (TTC)‐Evans Blue Double Staining

Twenty‐four hours after the reperfusion surgery, the LAD was re‐ligated. Hemostatic forceps were used to clamp the ascending aorta, and 0.5 mL of 1% Evans blue dye was slowly injected proximally into the ascending aorta. Once the dye was distributed throughout, the heart was quickly excised and frozen. The hearts were then cut into five consecutive 2 mm‐thick slices from the apex upward and stained with 1% TTC at 37°C in the dark for 15 min. The nonischemic region of the left ventricular area (LV) appeared blue, the ischemic size (IS) appeared white, and the area at risk (AAR) retained its original red color. The infarct size ratio was calculated by dividing the IS by AAR (IS/AAR). The area at risk ratio was calculated by dividing the area at risk by the left ventricular area (AAR/LV).

### 2.5 Histological and Immunostaining Analysis

After euthanizing the rats, their hearts were harvested. The hearts were washed with phosphate‐buffered saline (PBS) and fixed with 4% (w/v) paraformaldehyde (PFA) for 24 h, followed by dehydration with gradient concentrations of sucrose. The heart tissue was embedded in optimal cutting temperature, and continuous 8‐µm‐thick sections were prepared using a cryostat. Sections were stained with hematoxylin and eosin (HE) and Masson's trichrome staining according to the manufacturer's instructions. Wheat germ agglutinin staining (WGA) was performed using fluorescein isothiocyanate‐labeled probes (W11261, Thermo Fisher Scientific). Sheet samples were fixed with 4% PFA for 0.5 h, and then frozen sections were prepared for immunostaining.

For immunostaining, sections were incubated in blocking buffer (PBS containing 5% goat serum, 5% donkey serum, and 3% BSA) at room temperature for 2 h and permeabilized with 0.5% Triton X‐100. The sections were then incubated with primary antibodies at 4°C overnight. After washing with PBS, the sections were incubated with secondary antibodies at room temperature for 1 h. After counterstaining with 2‐(4‐amidinophenyl)‐1H‐indole‐6‐carboxamidine (DAPI, D523, Dojindo, Kumamoto, Japan), the sections were observed under a fluorescence microscope (BZ‐X800; KEYENCE, Japan) and a confocal microscope (SpinSR10, Olympus, Japan). The following antibodies were used: anti‐vWF (1:50, ab6994, Abcam), anti‐SMA (1:500, A2547, SIGMA), anti‐UCP‐1 (1:500, ab23841, Abcam), anti‐cTnT (1:500, sc‐20025, Santa Cruz Biotechnology) and α‐Tubulin (T9026, Sigma–Aldrich).

### 2.6 Cell Culture and Treatments

Human iPSC‐derived cardiomyocytes (iPSC‐CMs) were obtained as previously described [23]. Briefly, iPSCs (253G1, Riken, Tsukuba, Japan) were cultured in primate embryonic stem cell medium with basic fibroblast growth factor (bFGF) at 37°C using mitomycin C‐treated mouse embryonic fibroblasts as feeder cells. Cardiomyogenic induction was performed in the StemPro 34 medium supplemented with l‐glutamine, ascorbic acid, and 1‐thioglycerol. iPSCs were dissociated using Accmax and induced in a bioreactor with BMP4, activin A, bFGF, VEGF, and the small molecules IWR‐1 and IWP‐2, following a specific timeline. Human umbilical vein endothelial cells (HUVECs) were obtained from ATCC (CRL‐1730) and cultured in Endothelial Cell Basal Medium‐2 (CC‐3156, Lonza, Switzerland) supplemented with Endothelial Growth Medium‐2 SingleQuots Supplements (CC‐4176, Lonza, Switzerland). No patient or participant consent is required for this study.

For hypoxia/reoxygenation (H/R) treatment, iPSC‐CMs (5 × 10^5^ cells/cm^2^) were cultured in serum‐free, low‐glucose (0.3 g/L) DMEM under hypoxic conditions in a tri‐gas incubator (MINICell‐35, Waken, Japan) with 94% N_2_, 5% CO_2_, and 1% O_2_ for 12 h. The iPSC‐CMs were then re‐oxygenated by culturing in DMEM containing 10% FBS and 1% penicillin/streptomycin in an incubator with 5% CO_2_ and 95% air for an additional 12 h. HUVECs undergoing H/R treatment were cultured in basal medium during the 12 h hypoxia phase, followed by culture in basal medium supplemented with supplements during the 12 h reoxygenation phase, unless otherwise specified. In the H/R+BA group, regular medium was replaced with BA sheets‐conditioned medium during the reoxygenation phase. In the H/R+BA+AST1306 group, AST1306 (E0822, SELLECK) was added to the H/R+BA treatment during reoxygenation to achieve a final concentration of 0.8 nM, with no additional treatments applied.

### 2.7 Cell Viability Assay

The viability of the cardiomyocytes was assessed using a CCK‐8 assay kit (CK04, Dojindo, Kumamoto, Japan) according to the manufacturer's instructions. In brief, cells were seeded in 96‐well plates at a density of 5 × 10^5^ cells/cm^2^ (100 µL per well) and subjected to the above treatments. Subsequently, 10 µL of CCK‐8 solution was added to each well, and the cells were incubated for 1 h. Finally, absorbance was measured at 450 nm using a microplate reader (Synergy HTX, BioTek, USA).

### 2.8 Muscle Motion Analysis

Cardiomyocyte beating was recorded using a microscope (IX70, Olympus, Japan), and the ImageJ plugin MUSCLEMOTION was used to quantify cardiomyocyte contraction and relaxation. The beat frequency, maximum contraction velocity, maximum relaxation velocity, and contraction amplitude were calculated and compared as previously reported [24].

### 2.9 TUNEL Staining

As previously described [23], the apoptosis rate was assessed using a TUNEL staining kit (C10247, Thermo Fisher Scientific) according to the manufacturer's instructions. Samples were observed under a confocal microscope (SpinSR10, Olympus, Japan). ImageJ software was used to calculate the percentage of apoptotic nuclei by dividing the number of TUNEL‐stained nuclei by the total number of DAPI‐positive nuclei.

### 2.10 Western Blotting

Total protein was extracted by lysing the cells in RIPA buffer containing phosphatase and protease inhibitors. Nuclear proteins were extracted using NE‐PER Nuclear and Cytoplasmic Extraction Reagents (78833, Thermo Fisher Scientific) according to the manufacturer's instructions. Proteins were then separated by Sodium Dodecyl Sulfate‐Polyacrylamide Gel Electrophoresis (SDS‐PAGE) and transferred onto methanol‐treated 0.22 µm PVDF membranes. The membranes were then blocked with 5% TBST containing nonfat milk and incubated with the primary antibody overnight at 4°C. After three washes with TBST, the membranes were incubated with the secondary antibody at room temperature for 2 h. Protein bands were visualized and analyzed using a chemiluminescence (ECL) detection system (Image Lab, Bio‐Rad). The following primary antibodies were used: Actin (sc‐1616, Santa Cruz), ACSL4 (22401‐1‐AP, Proteintech), NRF2 (20733, CST), PI3K (4255, 4257, CST), p‐PI3K (4228, CST), AKT (4691S, CST), p‐AKT (4060T, CST), xCT (26864‐1‐AP, Proteintech), GPX4 (67763‐1‐Ig, Proteintech), TfR (10084‐2‐AP, Proteintech), ErbB4 (ab32375, Abcam), p‐ErbB4 (ab61059, Abcam), HO‐1 (ab68477, Abcam), Cleaved caspase‐3 (9661, CST), Caspase‐3 (9662, CST), Bcl‐2 (ab196495, Abcam), Bax (sc‐493, Santa Cruz), Lamin A/C (sc‐7292, Santa Cruz), NRG4 (11206‐1‐AP, Proteintech) and α‐Tubulin (T9026, Sigma–Aldrich).

### 2.11 RNA Sequencing

RNA sequencing was performed on the cardiomyocytes from the H/R+BA and H/R groups. Library preparation was conducted using the TruSeq Stranded mRNA Sample Preparation Kit (Illumina, San Diego, CA), following the manufacturer's instructions. Sequencing was performed on an Illumina NovaSeq 6000 platform using 100 bp paired‐end reads. Raw sequencing reads were trimmed using Trimmomatic v0.39 and mapped to the human reference genome, GRCh38. p5, using HISAT2 v2.1.0. Gene expression was quantified using featureCounts v2.0.6, and differential gene expression was analyzed using Cuffdiff v2.2.1. We used KOBAS (http://bioinfo.org/kobas) and Bioinformatics—a free online platform for data analysis and visualization [25] (http://www.bioinformatics.com.cn)—to analyze the differentially expressed genes (DEGs). The raw RNA sequencing data analyzed in this study have been deposited in the Gene Expression Omnibus under accession number GSE309984, which can be accessed at https://www.ncbi.nlm.nih.gov/geo/.

### 2.12 Intracellular ROS Assay

Intracellular ROS were detected using a DCFDA/H2DCFDA assay kit (ab113851, Abcam) following the manufacturer's instructions. Briefly, 1× wash buffer was prepared, and the cells were rinsed twice. The cells were incubated with the DCFDA solution for 45 min at 37°C in the dark. After incubation, the cells were washed twice with 1× buffer. Live cell microscopy was performed using a microscope (SpinSR10, Olympus, Japan) with a filter set appropriate for fluorescein to compare the fluorescence intensity across different groups.

### 2.13 Mitochondrial Membrane Potential Assay

The mitochondrial membrane potential (ΔΨm) was detected using the JC‐1 MitoMP Detection Kit (MT09, Dojindo, Japan), following the manufacturer's instructions. Briefly, the JC‐1 working solution and imaging buffer were prepared. An equal volume of the JC‐1 working solution was added directly to the medium. The cells were then incubated for an additional 30 min, the supernatant was discarded, and the cells were washed twice with HBSS. After adding imaging buffer, the cells were observed at excitation wavelengths of 488 nm (green) and 594 nm (red) using a fluorescence microscope (SpinSR10, Olympus, Japan).

### 2.14 Intracellular Fe^2+^ (FerroOrange) Assay

Intracellular Fe^2^^+^ was detected using the FerroOrange kit (F374, Dojindo, Japan) following the manufacturer's instructions. Briefly, a working solution of FerroOrange was prepared. The supernatant was discarded, and the cells were washed thrice with HBSS. We incubated the cells with the FerroOrange working solution for 30 min at 37°C. The cells were then observed under a fluorescence microscope, and images were captured (SpinSR10, Olympus, Japan).

### 2.15 TEM Assay

TEM (HT‐7800; Hitachi, Tokyo, Japan) was used to observe the ultrastructure of mitochondria in cardiomyocytes. TEM samples were prepared as follows: cell samples were fixed with 2.5% glutaraldehyde for 120 min, followed by postfixation with 1% osmium tetroxide for 90 min. The samples were then dehydrated in a graded ethanol series (50%–100%) and propylene oxide. Subsequently, tissues were embedded in epoxy resin, sectioned using an ultramicrotome (Ultracut E; Reichert‐Jung, Vienna, Austria), and stained with uranyl acetate and lead citrate. Mitochondrial damage was assessed in each group using the Flameng score [8]. Flameng scores were compared among groups using the Kruskal–Wallis test, followed by Dunn's post‐hoc analysis. Data are shown as violin plots with medians and interquartile ranges, and individual data points are overlaid.

### 2.16 Mitochondrial Respiration Assay

As previously described [26], mitochondrial respiration was assessed using a Seahorse XF96 Extracellular Flux Analyzer (Agilent Technologies, Carlsbad, CA). Cells were seeded in XF96 microplates (Agilent Technologies, Carlsbad, CA) and treated before the assay. Before measurement, the culture medium was replaced with Seahorse XF Base Medium (Agilent Technologies, Carlsbad, CA) supplemented with 1 mM sodium pyruvate, 2 mM glutamine, and 10 mM glucose. During the Mito Stress Test, mitochondrial inhibitors were sequentially added in the following order: 1 µM oligomycin, 3.5 µM 4‐(trifluoromethoxy)phenylhydrazone (FCCP; Seahorse Bioscience, Billerica, MA), and a combination of 0.5 µM rotenone and 0.5 µM antimycin A.

### 2.17 Wound Healing Assay

HUVECs were cultured in a culture dish to form a confluent monolayer. A scratch was made using a pipette tip to create a wounded area. Images of the scratched areas were captured at 0, 12, and 24 h using a microscope. The percentage of wound closure for each group was calculated using the ImageJ software and compared.

### 2.18 Transwell Assay

Briefly, 1 × 10^4^ HUVECs were seeded in the upper chamber of a Transwell insert. Cells were cultured in basal medium in the upper chamber and in basal medium supplemented in the lower chamber. For the H/R treatment, both the upper and lower chambers were filled with basal medium during the 12‐h hypoxia phase. After 12 h of reoxygenation, the lower chamber medium was replaced with supplemented basal medium. In the H/R+BA group, the lower chamber medium was replaced with BA sheets‐conditioned medium supplemented during the reoxygenation phase. The cells were fixed with 4% PFA, and the cells remaining on the upper side of the insert were removed using a cotton swab. The migrated cells on the lower side were stained with crystal violet and counted under a microscope (BZ‐X800; KEYENCE, Japan).

### 2.19 In Vitro Tube Formation Assay

Briefly, a 96‐well plate was precoated with Matrigel (356234, Corning, NY), and HUVECs were seeded at a density of 3 × 10 cells/well. The Control group was cultured in basal medium, whereas the treatment group was cultured for 4 h in BA sheets‐conditioned medium without supplementation. Images were captured under a brightfield microscope (CKX53, OLYMPUS, Japan), and the results were analyzed using the Angiogenesis Analyzer tool in ImageJ software [27].

### 2.20 siRNA Transfection

BA sheets were transfected with NRG4‐specific siRNA (siNRG4) or negative control siRNA(siNC) using Lipofectamine RNAiMAX Transfection Reagent (13778030, Thermo Fisher Scientific) according to the manufacturer's instructions. Briefly, siRNA and Lipofectamine RNAiMAX were separately diluted in Opti‐MEM I Reduced Serum Medium (31985070, Thermo Fisher Scientific), combined, and incubated at room temperature for 20 min to allow complex formation. The transfection mixture was then added to BA sheets cultured in antibiotic‐free medium. After 48 h, BA sheets were collected for subsequent experiments. The sequences of siRNAs and siNC are listed in Table S1.

### 2.21 Enzyme‐Linked Immunosorbent Assay (ELISA) Assay

The concentration of NRG4 in the conditioned medium was measured using a rat NRG4 ELISA kit (ER1739, FineTest, Wuhan, China) according to the manufacturer's instructions. Briefly, conditioned medium samples were collected and subjected to ELISA analysis following the provided protocol. Absorbance was measured at 450 nm with wavelength correction at 570 nm using a microplate reader (Synergy HTX, BioTek, USA).

### 2.22 Statistical Analysis

All quantitative data are expressed as mean ± SD and were analyzed using GraphPad Prism 9.5.0 (GraphPad Software, USA). The differences between the two groups for normally distributed variables were assessed using Student's *t*‐test. For comparisons among three or more groups, a one‐way ANOVA followed by Tukey's post‐hoc test (with homogeneity of variance) was performed. Ordinal data are expressed as medians with interquartile ranges and were analyzed using the Kruskal–Wallis test followed by Dunn's multiple comparisons test. Statistical significance was set at *p* < 0.05, with significance levels defined as ^*^ *p* < 0.05, ^**^ *p* < 0.01, ^***^ *p* < 0.001, and ^****^ *p* < 0.0001.

## 3 Results

### 3.1 Establishment and Characterization of BA Sheets

The establishment of BA sheets and the subsequent transplantation procedures are shown in Figure S1a. The preadipocytes were induced to differentiate into mature BAs. As shown in the brightfield images, morphological changes were progressively observed from day 0–6 during differentiation, characterized by increased intracellular lipid accumulation (Figure S1b). Oil Red O staining on day 6 confirmed the presence of abundant lipid droplets, which appeared as multiple small droplets surrounding a centrally located nucleus, which is a morphological hallmark of BAs (Figure S1c). Immunofluorescence analysis further demonstrated strong expression of the brown adipocyte‐specific marker UCP‐1 (Figure S1d,e), confirming the successful induction of functional BAs.

Next, to construct transplantable BA sheets, we combined BAs with poly (lactic‐co‐glycolic acid) (PLGA) fibers, a biodegradable cell carrier developed by our group [2, 23, 28, 29] (Figure S1f), and tested different seeding densities (1 × 10^6^, 3 × 10^6^, 5 × 10^6^, and 7 × 10^6^ cells/cm^2^) (Figure S1g). Cross‐sectional cryosections revealed that an increased seeding density correlated with thicker and more compact sheets. Immunostaining of the cryosections confirmed that BAs within the sheets maintained UCP‐1 expression, indicating that BAs differentiation was preserved after sheet formation. Quantitative analysis showed a density‐dependent increase in the sheet's thickness. However, at the highest seeding density (7 × 10^6^ cells/cm^2^), we observed a reduction in cell density within the inner sheets region, likely due to insufficient nutrient diffusion and reduced cell viability. Therefore, we selected 5 × 10^6^ cells/cm^2^ as the optimal density for subsequent experiments.

### 3.2 BA Sheets Alleviate Cardiac Dysfunction Following Myocardial I/R Injury

**FIGURE 1:**
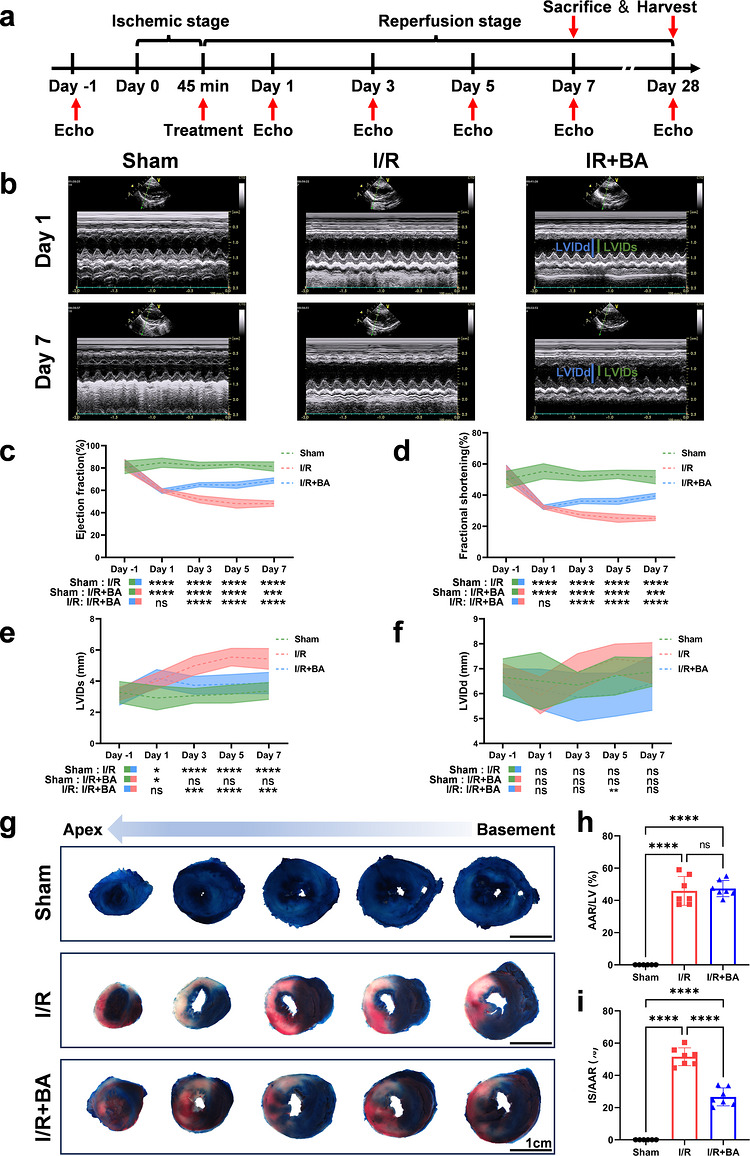
BA sheets alleviate cardiac dysfunction following myocardial I/R injury in rats. (a) Schematic diagram showing the rat myocardial I/R injury model and subsequent procedures. (b) Representative M‐mode echocardiography images of the Sham, I/R, and I/R+BA groups on days 1 and 7 postreperfusion. (c–f) Heart function represented as LVEF (c), LVFS (d), left ventricle internal diameter at end‐systole (LVIDs) (e), and left ventricle internal diameter at end‐diastole (LVIDd) (f) at baseline (day ‐1) and days 1, 3, 5, and 7 post I/R (Sham: n = 7; I/R: n = 8; I/R+BA: n = 8). (g) Triphenyltetrazolium chloride (TTC) blue double staining of heart sections was performed 24 h after reperfusion. IS is shown in white, the AAR in red, and the nonischemic region of the LV in blue (scale bar = 1 cm). (h,i) Quantification of AAR/LV (h) and IS/AAR (i) ratios (Sham: n = 6; I/R: n = 7; I/R + BA: n = 7). Data are presented as mean ± SD. Statistical significance was determined using one‐way ANOVA, followed by Tukey's post‐hoc test. ^*^ *p* < 0.05; ^**^ *p* < 0.01; ^***^ *p* < 0.001; ^****^ *p* < 0.0001; ns, not significant.

To evaluate the effects of BAs on cardiac systolic and diastolic functions after myocardial I/R injury, we transplanted BA sheets onto the surface of the injured myocardium in rats subjected to I/R injury (Figure S1h). Cardiac function was evaluated using 2D echocardiography conducted 1 day prior to surgery (day −1) and on days 1, 3, 5, and 7 postreperfusion (Figure 1a,b). On day −1, no significant differences were noted in the left ventricular ejection fraction (LVEF) or left ventricular fractional shortening (LVFS) among the Sham, I/R, and I/R+BA groups. Marked decreases in LVEF and LVFS were observed on day 1 in both ischemia groups, confirming the successful establishment of the I/R model. However, no significant differences were detected between the two ischemia groups at this stage (Figure 1c,d). The LVEF in the I/R+BA group was significantly higher than that in the I/R group on days 3, 5, and 7 after treatment (Figure 1c). Similarly, LVFS in the I/R+BA group was significantly higher than that in the I/R group starting 1 day after treatment, and this trend closely mirrored that of LVEF (Figure 1d). The LVIDs in the I/R group were significantly higher than those in the I/R+BA group on day 1 after treatment, indicating worse systolic function (Figure 1e). In contrast, the LVIDd was not significantly different between the two groups (Figure 1f).

In addition, to assess longer‐term effects and enhance specificity, the observation period was extended to 28 days, and sheets constructed from brown preadipocytes without differentiation induction (I/R+PreBA) and sheets derived from white adipocytes (I/R+WA) were also included (Figure S2a–c). LVEF and LVFS in the I/R+PreBA and I/R+WA groups did not show improvement and followed a trend like the I/R group, remaining significantly lower than those in the I/R+BA group on days 3, 5, and 7 after treatment. LVEF and LVFS stabilized after days 5–7, and this trend was maintained up to day 28 (Figure S2d,e).

### 3.3 BA Sheets Reduce Infarct Size in the Myocardium

To investigate the effect of BA sheets on myocardial infarct size after I/R injury, continuous heart sections were collected from rats 24 h after the procedure and stained with triphenyltetrazolium chloride (TTC)/Evans Blue (Figure 1g). No significant differences were noted in the area at risk (AAR) to the nonischemic region of the left ventricle (AAR/LV) ratio between the two ischemic groups (Figure 1h). However, the infarct size (IS) to AAR (IS/AAR) ratio was significantly elevated in both ischemic groups, whereas it was notably lower in the I/R+BA group than in the I/R group (Figure 1i), indicating that BA sheets treatment effectively reduced the myocardial infarct size.

### 3.4 BA Sheets Reduce Myocardial Fibrosis and Inhibit Ventricular Remodeling

**FIGURE 2:**
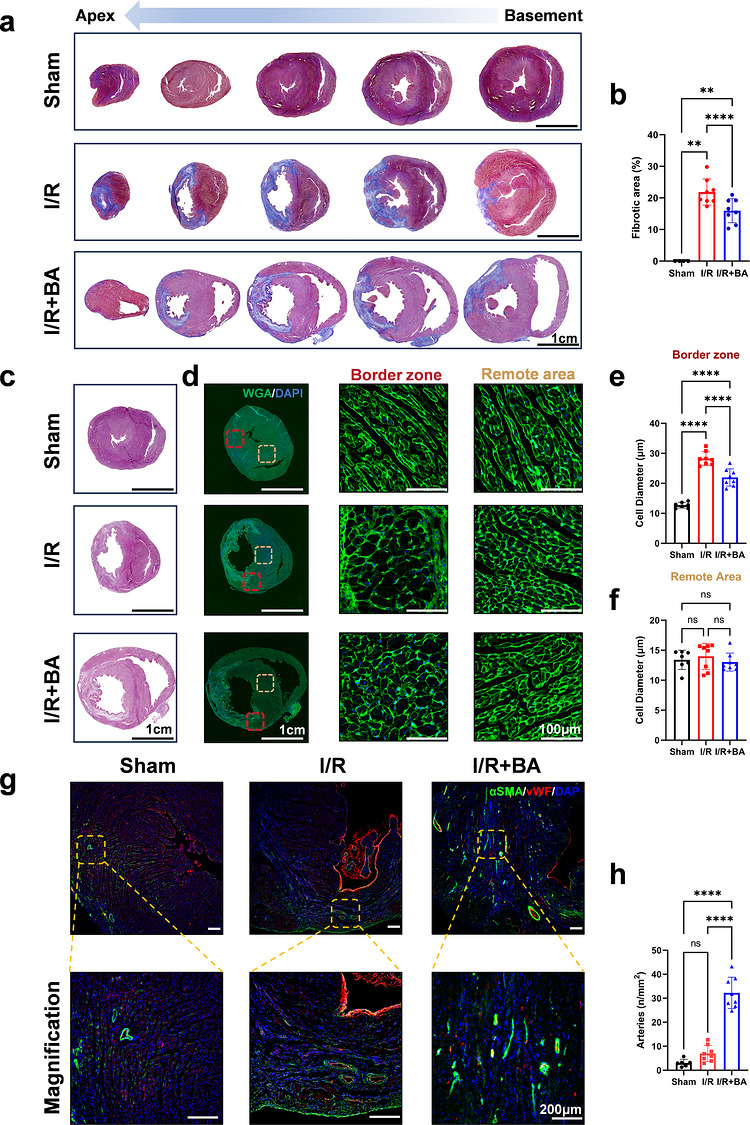
BA sheets attenuate myocardial fibrosis, suppress ventricular remodeling, and enhance angiogenesis following I/R injury. (a) Masson's trichrome staining of heart sections 7 days post‐reperfusion. The fibrotic areas are stained blue (scale bar = 1 cm). (b) Quantification of fibrotic area as a percentage of the total myocardial area. (c) HE staining of heart sections (scale bar = 1 cm). (d) WGA staining of heart sections, highlighting cardiomyocyte membranes (green). Images are shown for each group, including original sections and magnified views of both the border (red box) and remote zones (yellow box). Original image scale bar = 1 cm; magnified view scale bar = 100 µm. (e,f) Quantitative analysis of cardiomyocyte diameter in the border zone (e) and remote zone (f). (g) Immunofluorescence images of the border zone stained for alpha smooth muscle actin (αSMA, green), von Willebrand factor (vWF, red), and nuclei (DAPI, blue) (scale bar = 200 µm). (h) Quantification of vascular density based on α‐SMA/vWF double‐positive structures. The same group sizes (Sham, n = 7; I/R, n = 8; I/R+BA, n = 8) were used for all quantifications. Data are presented as mean ± SD. Statistical significance was determined using a one‐way ANOVA followed by Tukey's post‐hoc test. ^**^ *p* < 0.01; ^****^ *p* < 0.0001; ns, not significant.

To investigate the mechanisms underlying the improvement in cardiac function, we examined the extent of cardiac fibrosis using Masson's trichrome staining (Figure 2a). Compared with the Sham group, the ischemic groups exhibited significant myocardial fibrosis on day 7 (Figure 2b). However, the fibrotic area in the I/R+BA group was significantly smaller than that in the I/R group, suggesting that BA sheets could inhibit myocardial fibrosis.

Next, we performed hematoxylin and eosin (HE) and wheat germ agglutinin (WGA) staining to assess ventricular remodeling and cardiomyocyte hypertrophy (Figure 2c). No significant differences were observed in the cardiomyocyte diameter (measured as the short axis across the nucleus) in the remote region among the groups (Figure 2d,e). However, in the border zone, the cardiomyocyte diameter in the ischemic groups was significantly larger than that in the Sham group, consistent with Masson's trichrome staining results. In contrast, the cardiomyocyte diameter in the I/R+BA group was smaller than that in the I/R group, suggesting that BA sheets reduced ventricular remodeling and suppressed cardiomyocyte hypertrophy.

To further evaluate the long‐term effects on cardiac remodeling, the above histological analyses were also performed at 28 days after I/R injury, with the I/R+PreBA and I/R+WA groups included. Both the I/R+PreBA and I/R+WA groups exhibited results similar to those of the I/R group at day 28, with no evidence of improvement, showing larger fibrotic areas and increased cardiomyocyte diameter in the border zone compared with the I/R+BA group (Figure S3a–f). These findings were consistent with the echocardiographic results.

### 3.5 BA Sheets Promote Angiogenesis in Host Myocardium

We performed immunofluorescence staining to evaluate vascular density in the border zone of the host myocardium (Figure 2f). Endothelial cells were identified by von Willebrand factor (vWF) staining and smooth muscle cells by alpha smooth muscle actin (αSMA) staining, while nuclei were counterstained with DAPI. The I/R+BA group had a higher vascular density than the I/R and Sham groups, whereas no significant difference was observed between the I/R and Sham groups (Figure 2g). These results suggest that BA sheets promote angiogenesis in the border zones of the infarcted hearts.

### 3.6 BA Sheets Enhance Endothelial Migration and Angiogenic Capacity In Vitro

To further explore the mechanism by which BA sheets promote angiogenesis, we assessed endothelial cell migration in human umbilical vein endothelial cells (HUVECs) using a wound healing assay. As shown in the brightfield images, wound closure was impaired in the hypoxia/reoxygenation (H/R) group compared to the Control group. At 12 h post injury, no significant differences were observed between the H/R and H/R+BA groups (Figure S4a,b). However, after 24 h, the BA sheets treatment markedly enhanced wound closure compared with H/R alone, as demonstrated by both representative images and quantitative analysis (Figure S4a,c).

Transwell migration assay performed after H/R injury showed that HUVECs migration was significantly reduced in the I/R group, whereas culture in BA sheets‐conditioned medium restored the migratory ability to a level comparable to that of the Control group (Figure S4d,e). To further assess the effect of BA sheets on the angiogenic capacity, we conducted a tube formation assay. HUVECs cultured in BA sheets‐conditioned medium formed more extensive capillary‐like structures than those cultured in basal medium, as shown in whole‐field and magnified brightfield images captured at 4 h (Figure S4f). Quantitative analysis revealed a significant increase in the branch number and total tube length in the conditioned medium group (Figure S4g,h).

### 3.7 BA Sheets Attenuate Endothelial Cell Death Following H/R Injury

To evaluate the protective effects of BA sheets against H/R‐induced endothelial injury, we assessed the viability and apoptosis of HUVECs. Live/dead staining revealed a substantial increase in cell death in the H/R and H/R+BAs groups compared to that in the Control group. However, BA sheets treatment during reoxygenation significantly preserved cell viability in the H/R+BA group compared to that in the H/R group (Figure S5a,b). Consistent with this, TUNEL staining demonstrated elevated apoptosis following H/R injury in the H/R and H/R+BAs groups compared to the Control group. Similarly, treatment with BA sheets significantly reduced the number of TUNEL‐positive cells, suggesting an anti‐apoptotic effect (Figure S5c,d).

### 3.8 BA Sheets Enhance Cardiomyocyte Survival, Restore Contraction and Relaxation Functions, and Inhibit Apoptosis Following H/R

**FIGURE 3:**
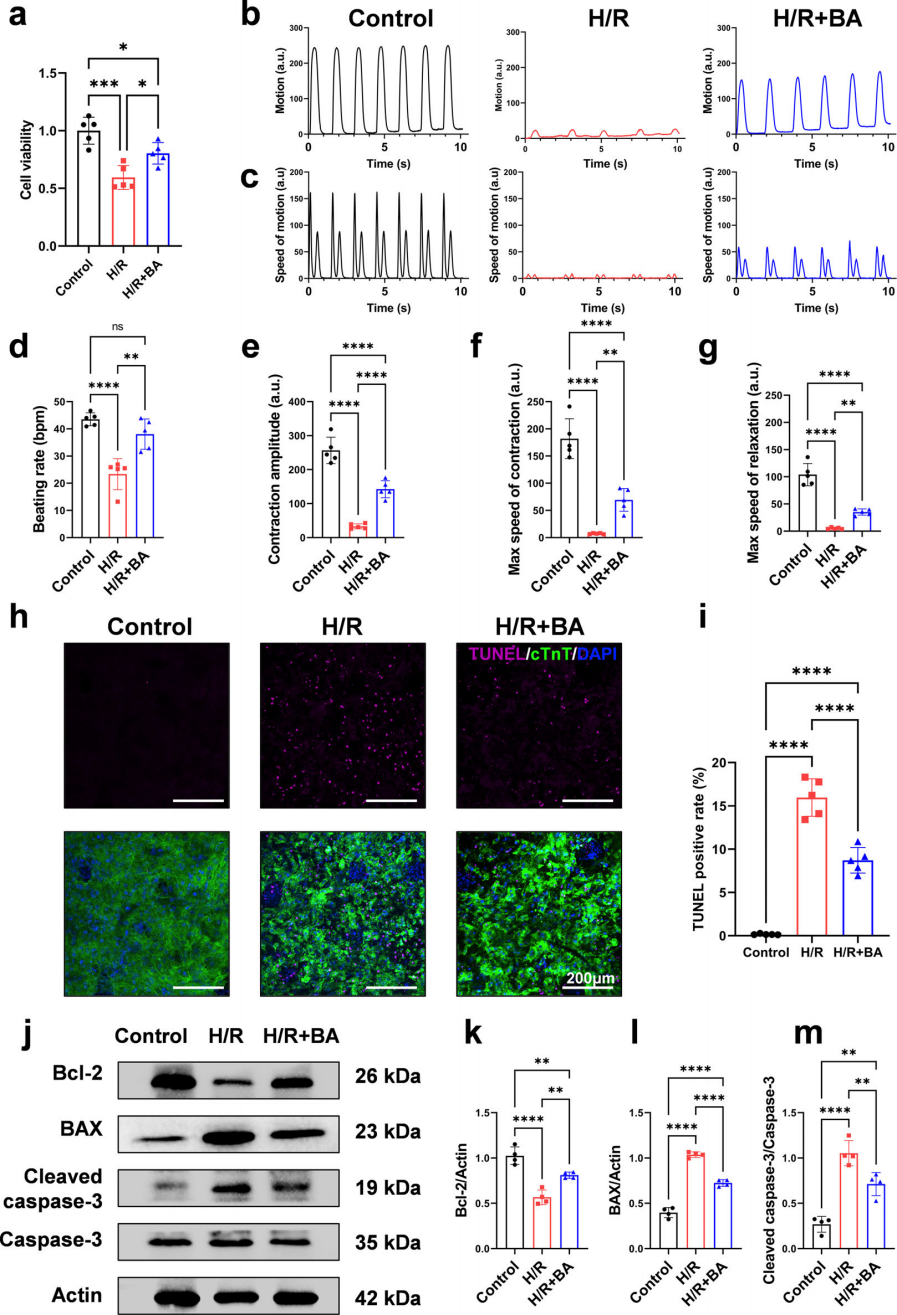
BA sheets mitigate cardiomyocyte injury caused by hypoxia/reoxygenation (H/R) injury. (a) Cell viability analysis of cardiomyocytes assessed by the CCK‐8 assay from Control, H/R, and H/R+BA groups (n = 5 per group). (b,c) Representative contraction amplitude traces (b) and contraction/relaxation velocity traces (c) of cardiomyocytes under different treatments. (d–g) Quantitative comparison of contraction frequency (bpm, d), contraction amplitude (e), maximum contraction velocity (f), and maximum relaxation velocity (g) in the Control, H/R, and H/R+BA groups (n = 5 per group). (h) Immunofluorescence images of cardiomyocytes for cTnT (green), TUNEL (violet), and nuclei (DAPI, blue) (scale bar = 200 µm). (i) Quantification of TUNEL‐positive cardiomyocytes from the Control, H/R, and H/R+BA groups (n = 5 per group). (j) Representative western blots of apoptosis‐related proteins, including Bcl‐2, Bax, Cleaved caspase‐3, and total caspase‐3. (k–m) Quantification of protein expression levels: Bcl‐2/Actin (k), BAX/actin (l), and cleaved caspase‐3/Caspase‐3 ratio (m) from the Control, H/R, H/R+BA groups (n = 4 per group). Data are presented as mean ± SD. Statistical significance was determined by one‐way ANOVA, followed by Tukey's post‐hoc test. ^*^ *p* < 0.05; ^**^ *p* < 0.01; ^***^ *p* < 0.001; ^****^ *p* < 0.0001; ns, not significant.

To elucidate the effects of BA sheets on cardiomyocytes subjected to H/R injury, we established an H/R model using human‐induced pluripotent stem cell‐derived cardiomyocytes (hiPS‐CMs) and assessed their functionality. In the CCK‐8 assay, cell viability was significantly decreased in the H/R and H/R+BA groups compared to the Control group. However, in the H/R+BA group, cell viability was markedly restored compared with that in the H/R group (Figure 3a).

The critical functions of cardiomyocytes are contraction and relaxation. We recorded videos (Videos S1–S3) and analyzed and visualized the beat patterns of the cardiomyocytes (Figure 3b,c). As shown in Figure 3d, the beat frequency was significantly reduced in the H/R group following hypoxia, whereas it was significantly higher in the H/R+BA group, which was not significantly different from that in the Control group. Similarly, the beating amplitude, maximum contraction velocity, and maximum relaxation velocity (Figure 3e–g) decreased in both hypoxia groups compared to the Control group. However, these parameters were significantly restored in the H/R+BA group compared with those in the H/R group. We further performed TUNEL staining (Figure 3h) to evaluate apoptosis. The apoptosis rate increased following hypoxia, but was notably reduced in the H/R+BA group compared to that in the H/R group (Figure 3i). In line with these findings, western blot analysis (Figure 3j) showed that the expression of the anti‐apoptotic protein Bcl‐2 (Figure 3k) was upregulated, while the pro‐apoptotic proteins Bax (Figure 3l) and Cleaved Caspase‐3 (Figure 3m) were downregulated in the H/R+BA group compared to the H/R group, which is consistent with previous studies [15].

### 3.9 Transcriptomic Profiling

**FIGURE 4:**
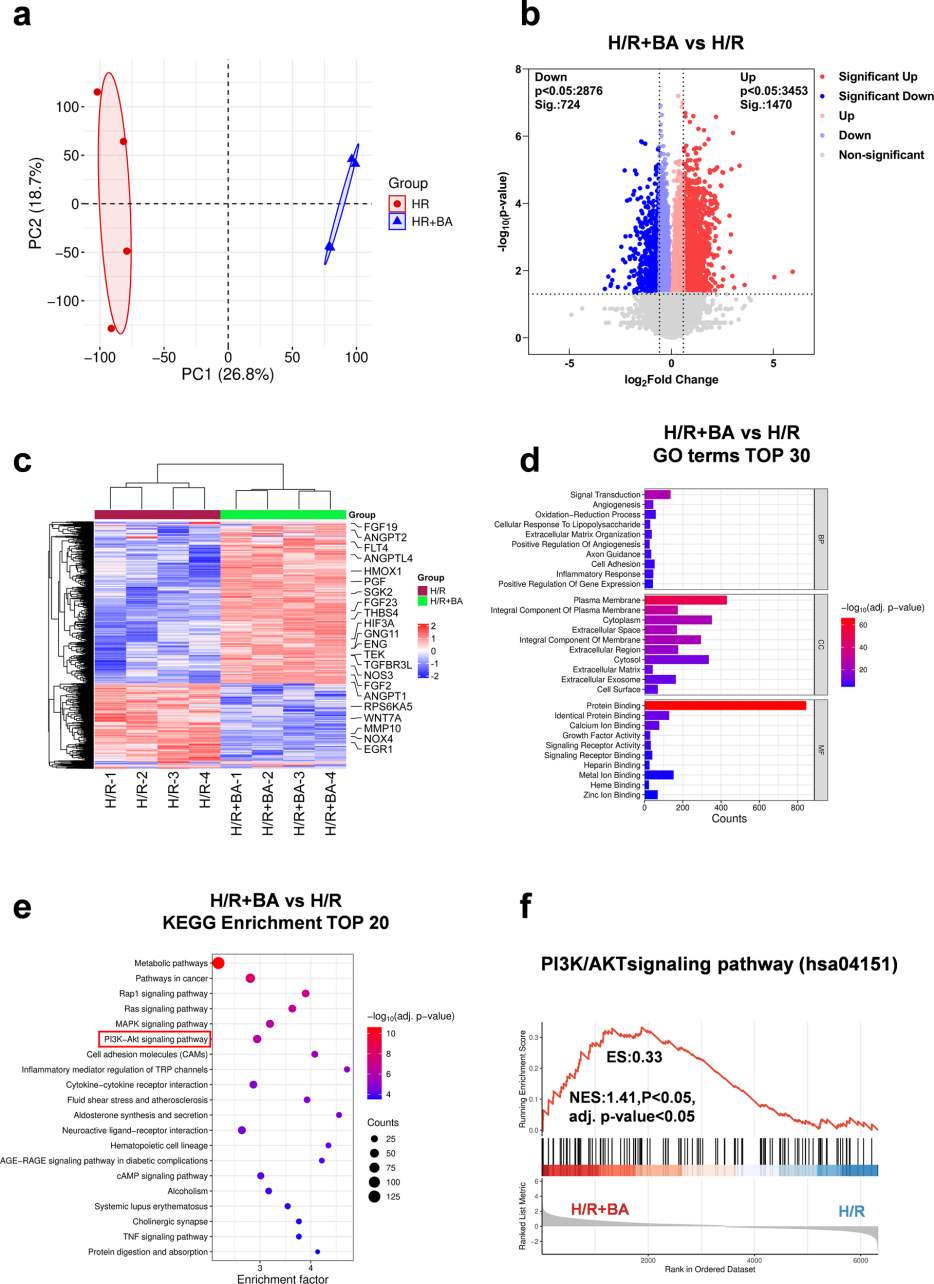
Transcriptomic profiling reveals regulatory effects of BA sheets on cardiomyocytes following H/R injury. (a) PCA showing clear separation of samples from the H/R and H/R+BA groups (n = 4 per group). (b) Volcano plot displaying the distribution of DEGs between H/R and H/R+BA groups. (c) Heatmap illustrating the hierarchical clustering of DEGs across the H/R and H/R+BA groups. (d) GO enrichment analysis showing the top 30 biological processes, cellular components, and molecular functions associated with the DEGs. (e) KEGG enrichment analysis highlighting the top 20 significantly enriched pathways. (f) Gene Set Enrichment Analysis (GSEA) indicates activation of the PI3K/AKT signaling pathway in the H/R+BA group.

To elucidate the molecular mechanisms underlying the protective effects of BA sheets on cardiomyocytes under H/R stress, we performed RNA sequencing of hiPS‐CMs from the H/R and H/R+BA groups. Principal component analysis (PCA) showed that samples from the H/R and H/R+BA groups were clearly separated along PC1 (accounting for 26.8% of the variance), indicating that the treatment induced changes in the overall gene expression patterns (Figure 4a). We detected 60,658 genes, and differential expression analysis visualized using a volcano plot identified 2194 differentially expressed genes (DEGs), of which 1470 were upregulated, and 724 were downregulated in the H/R+BA group (Figure 4b). Subsequently, hierarchical clustering of these DEGs was performed and visualized as a heatmap, which showed that samples within the same group exhibited highly similar expression patterns, whereas distinct differences were observed between groups, providing a reliable set of candidate genes for subsequent Gene Ontology (GO) and Kyoto Encyclopedia of Genes and Genomes (KEGG) enrichment analyses (Figure 4c). GO analysis highlighted biological processes, such as angiogenesis, oxidation–reduction, inflammatory response, extracellular matrix organization, and cell adhesion, along with molecular functions, such as protein and calcium ion binding (Figure 4d). KEGG enrichment analysis revealed significant enrichment of survival‐ and stress‐related pathways, including PI3K/AKT, MAPK, TNF signaling, cytokine–cytokine receptor interactions, and metabolic pathways (Figure 4e). These changes indicate enhanced cell survival and tissue repair. Gene Set Enrichment Analysis (GSEA) further confirmed the upregulation of the PI3K/AKT signaling pathway in the H/R+BA group (Figure 4f), suggesting its key role in BA sheets‐mediated cardioprotection.

BAs secrete bioactive factors that regulate systemic and local metabolic activities. Among these, neuregulin 4 (NRG4), a member of the epidermal growth factor family, is predominantly expressed in the peripheral tissues, with the highest abundance found in BAT, which has significant therapeutic potential [30]. NRG4 is a ligand of the ErbB4 receptor on the surface of target cells. Upon ligand binding, ErbB4 initiates a cascade of pro‐survival signaling events by activating downstream pathways, including PI3K/AKT, AMPK/mTOR, and STAT5 [30, 31, 32]. Based on these observations and our transcriptomic findings, we hypothesized that ErbB4 is a candidate through which BA sheets may exert cardioprotective effects by interacting with NRG4. The NRG4–ErbB4 interaction may activate the PI3K/AKT signaling pathway, potentially modulating the downstream NRF2/HO‐1 antioxidant axis. Given the established role of NRF2/HO‐1 signaling in mitigating ferroptosis, we speculated that this mechanism may contribute to enhanced cardiomyocyte survival under H/R conditions. The proposed model offers a potential molecular link between BA sheets‐derived paracrine signaling and the pro‐survival transcriptional reprogramming observed in our RNA sequencing data.

### 3.10 BA Sheets Can Alleviate Ferroptosis and Mitochondrial Dysfunction in H/R‐Stressed Cardiomyocytes in an ErbB4–Dependent Manner

**FIGURE 5:**
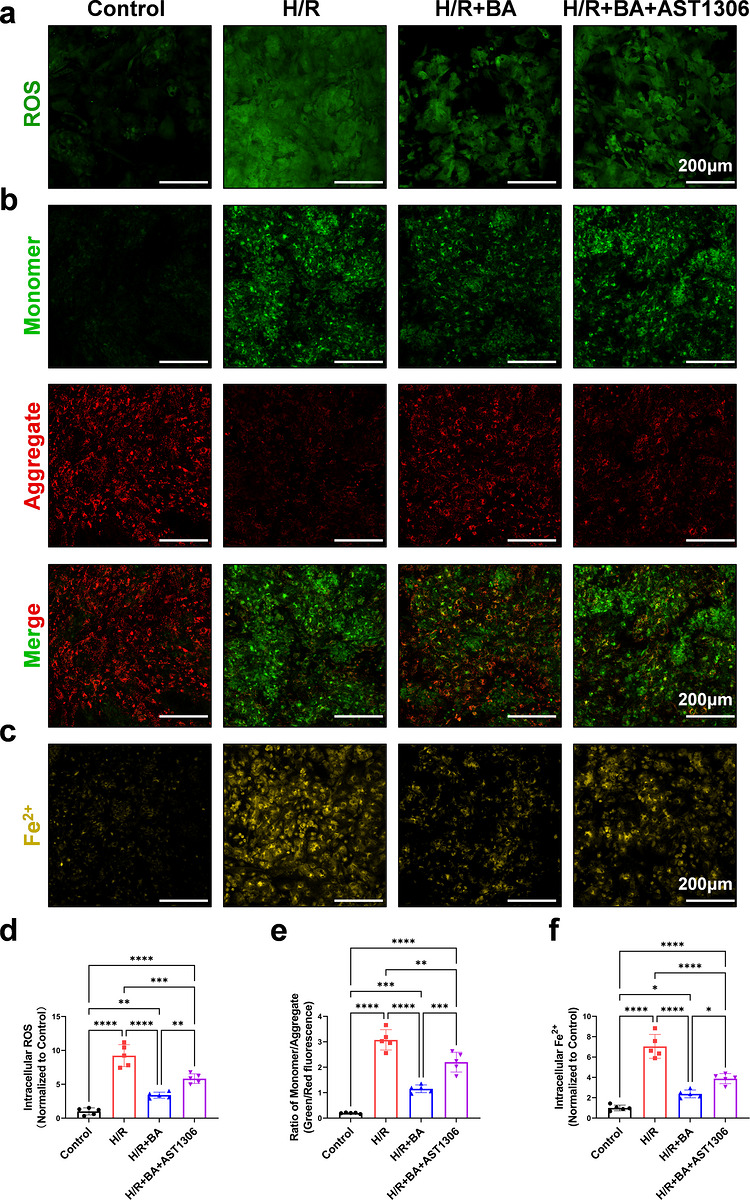
BA sheets alleviate ferroptosis‐related oxidative stress following H/R injury in cardiomyocytes in an ErbB4–dependent manner. (a) Representative fluorescence images of intracellular ROS in cardiomyocytes. (b) JC‐1 staining showing mitochondrial membrane potential (ΔΨm), where red fluorescence (aggregates) indicates polarized mitochondria and green fluorescence (monomers) indicates depolarized mitochondria. (c) Fluorescent images of intracellular ferrous iron (Fe^2+^) levels detected by FerroOrange fluorescence probe. (d–f) Quantification of fluorescence intensity for ROS (d), JC‐1 ratio (monomer/aggregate) (e), and intracellular Fe^2+^ (f), respectively, normalized to the Control group. The same group sizes (n = 5) and scale bars (200 µm) were used for all four groups (Control, H/R, H/R+BA, and H/R+BA+AST1306). Data are presented as mean ± SD. Statistical significance was determined by one‐way ANOVA followed by Tukey's post‐hoc test. ^*^ *p* < 0.05; ^**^ *p* < 0.01; ^***^ *p* < 0.001; ^****^ *p* < 0.0001.

To validate our hypothesis, we introduced AST1306, an inhibitor of ErbB4, into the H/R+BA group and examined ferroptosis‐related parameters, including ROS, mitochondrial membrane potential (ΔΨm), and intracellular ferrous iron (Fe^2+^) (Figure 5a–f). ROS levels were significantly elevated in cardiomyocytes subjected to H/R compared with those in the Control group (Figure 5a,d). Treatment with BA sheets effectively reduced ROS accumulation; however, this protective effect was attenuated by the addition of AST1306, resulting in a rebound in ROS levels. Similarly, ΔΨm, represented by the JC‐1 monomer/aggregate ratio (Figure 5b,e), and intracellular Fe^2+^ levels (Figure 5c,f) followed the same trend as ROS, showing significant impairment after H/R that was mitigated by BA sheets but partially reversed by AST1306. These findings indicated that BA sheets confer cardioprotection by inhibiting ferroptosis in an ErbB4–dependent manner.

**FIGURE 6:**
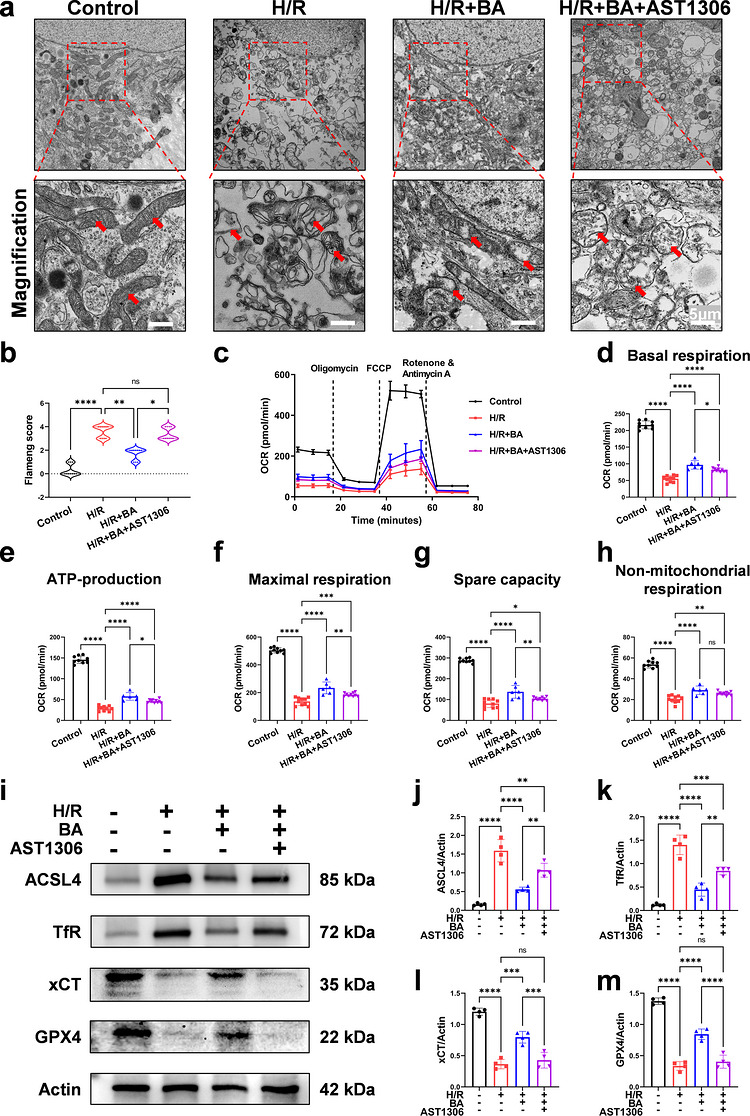
BA sheets preserve mitochondrial integrity and suppress ferroptosis in H/R‐injured cardiomyocytes in an ErbB4–dependent manner. (a) Representative transmission electron microscopy (TEM) images of cardiomyocyte mitochondria. The upper panels show the original field, and the lower panels display magnified views of the boxed regions. Red arrows indicate mitochondria, scale bar = 5 µm. (b) Analysis of mitochondrial ultrastructural damage based on the Flameng scoring criteria (n = 10 per group). Data are expressed as medians with interquartile ranges and were analyzed using the Kruskal–Wallis test followed by Dunn's multiple comparisons test. (c) Seahorse XF analysis of mitochondrial oxygen consumption rate profiles in cardiomyocytes. (d–h) Quantification of basal respiration (d), ATP production (e), maximal respiration (f), spare capacity (g), and nonmitochondrial respiration (h). (i) Representative western blots of ferroptosis‐related proteins (ACSL4, TfR, xCT, and GPX4). (j–m) Densitometric quantification of ACSL4 (j), TfR (k), Xct (l), and GPX4 (m) expression levels normalized to those of the Control group (n = 4 per group). Data are presented as mean ± SD. Statistical significance was determined by one‐way ANOVA, followed by Tukey's post‐hoc test. ^*^ *p* < 0.05; ^**^ *p* < 0.01; ^***^ *p* < 0.001; ^****^ *p* < 0.0001; ns, not significant.

To further elucidate the protective mechanism of the BA sheets, we examined their mitochondrial ultrastructure using transmission electron microscopy (TEM). H/R induces pronounced mitochondrial damage, including the disruption of membranes, loss of cristae, and swelling. Flameng scores revealed significantly elevated mitochondrial injury in the H/R group, which was mitigated by the BA sheets. However, this protective effect was partially reversed by co‐treatment with the ErbB4 inhibitor AST1306 (Figure 6a,b). Mitochondrial respiratory function was evaluated using Seahorse XF analysis (Figure 6c–h). H/R significantly impaired mitochondrial function, as reflected by reductions in basal respiration, ATP production, maximal respiration, spare‐respiratory capacity, and nonmitochondrial respiration (Figure 6d–h). Treatment with the BA sheets effectively restored these functional parameters. Similarly, co‐treatment with AST1306 partially blocked recovery, except for nonmitochondrial respiration. These results indicated that the mitochondrial protective effects of BA sheets depend on ErbB4 activation.

To validate the impact of ferroptosis at the molecular level, we analyzed key ferroptosis‐related proteins. Western blot analysis showed that H/R upregulated the ferroptosis‐promoting proteins ACSL4 and TfR and downregulated the ferroptosis‐inhibitory proteins xCT and GPX4 (Figure 6i–m). The BA sheets reversed these changes. However, this modulation was markedly attenuated by AST1306, further supporting the regulatory role of ErbB4 in ferroptosis. These results confirm that the BA sheets exert cardioprotective effects by mitigating ferroptosis and preserving mitochondrial structure and function via an ErbB4–dependent mechanism.

### 3.11 BA Sheets Protect Cardiomyocytes Against Ferroptosis by Modulating ErbB4 and Activating the PI3K/AKT and NRF2/HO‐1 Signaling Pathways Following H/R Injury

**FIGURE 7:**
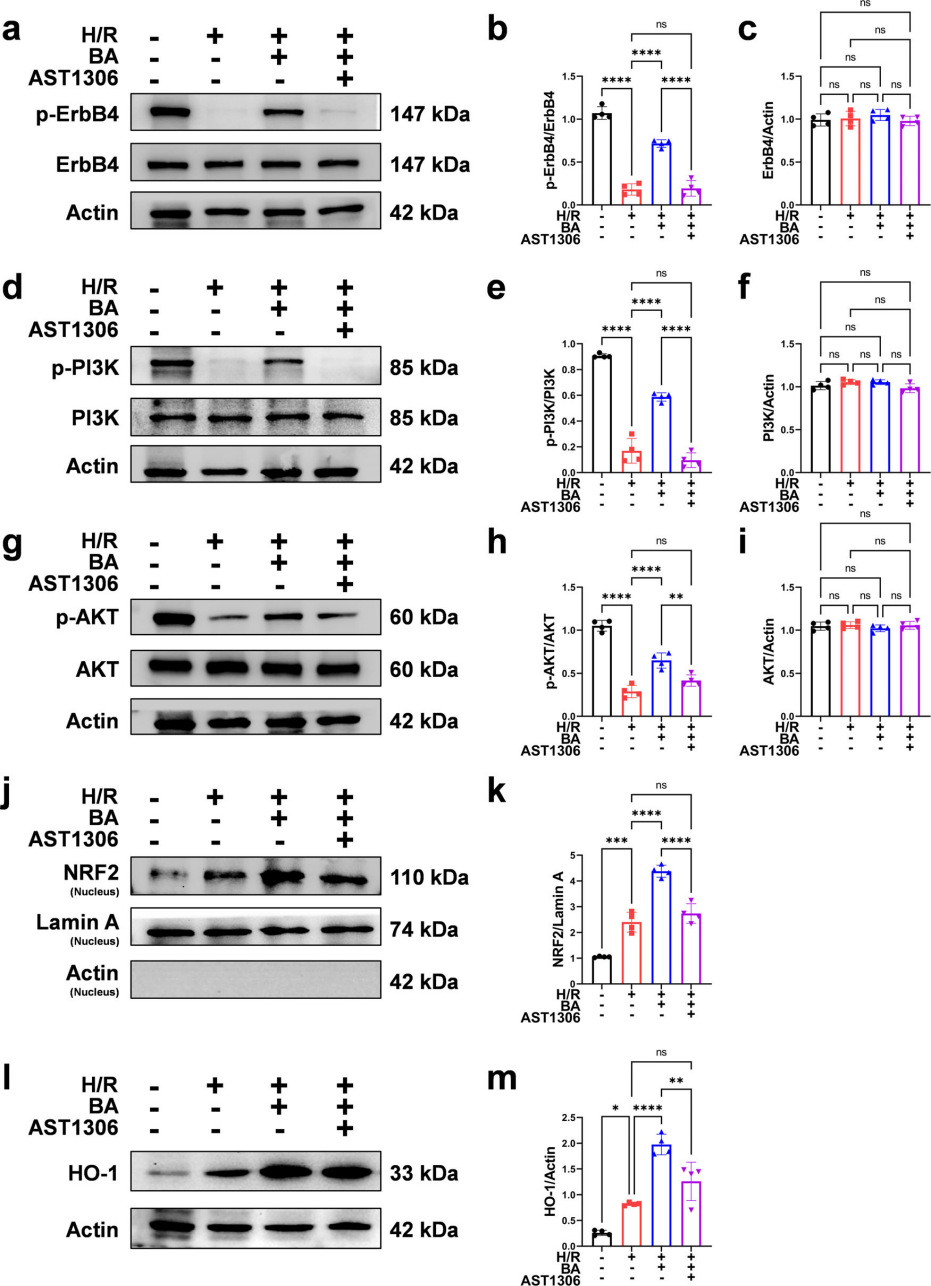
BA sheets protect cardiomyocytes against ferroptosis by activating ErbB4 and downstream PI3K/AKT and NRF2/HO‐1 signaling pathways following H/R injury. (a) Representative western blots of total and phosphorylated ErbB4. (b,c) Densitometric quantification of total and phosphorylated ErbB4 normalized to actin (n = 4 per group). (d) Representative western blots of total and phosphorylated PI3K levels. (e,f) Densitometric quantification of total and phosphorylated PI3K normalized to actin (n = 4 per group). (g) Representative western blots of total and phosphorylated AKT levels. (h,i) Densitometric quantification of total and phosphorylated AKT normalized to actin (n = 4 per group). (j) Representative western blots of NRF2 (nuclear fraction; Lamin A is loading control). (k) Densitometric quantification of NRF2 normalized to the Control group (n = 4 per group). (l) Representative western blot of HO‐1. (m) Densitometric quantification of HO‐1 normalized to that of the Control group (n = 4 per group). Data are presented as mean ± SD. Statistical significance was determined by one‐way ANOVA, followed by Tukey's post‐hoc test. ^*^ *p* < 0.05; ^**^ *p* < 0.01; ^***^ *p* < 0.001; ^****^ *p* < 0.0001; ns, not significant.

To elucidate the molecular mechanism by which BA sheets suppress ferroptosis in cardiomyocytes after H/R injury, guided by our previous transcriptomic analysis, we examined the key protein ErbB4 and its downstream antioxidant pathways, PI3K/AKT and NRF2/HO‐1. Western blot analysis revealed that H/R significantly decreased the phosphorylation levels of ErbB4, PI3K, and AKT (Figure 7a–i), indicating the suppression of this signaling pathway. Conversely, nuclear NRF2 level was increased in the H/R group (Figure 7j,k), which was consistent with a cellular compensatory response to oxidative stress and ferroptosis. HO‐1 expression, a downstream antioxidant enzyme regulated by NRF2, also showed an upward trend (Figure 7l,m), reflecting the activation of endogenous antioxidant defenses. Treatment with BA sheets‐conditioned medium significantly restored the phosphorylation of ErbB4, PI3K, and AKT, further promoting NRF2 nuclear translocation and enhancing HO‐1 expression. These results suggest that BA sheets activate the ErbB4 and PI3K/AKT signaling pathways, which facilitate NRF2‐mediated antioxidant responses to mitigate ferroptosis. Furthermore, co‐treatment with the ErbB4 inhibitor AST1306 partially abolished the phosphorylation of ErbB4, PI3K, and AKT and reduced nuclear NRF2 accumulation and HO‐1 expression, confirming the crucial role of ErbB4 activation in this protective mechanism.

### 3.12 BA Sheets Suppress Ferroptosis via NRG4–Dependent ErbB4 Activation in Cardiomyocytes Following H/R Injury

**FIGURE 8:**
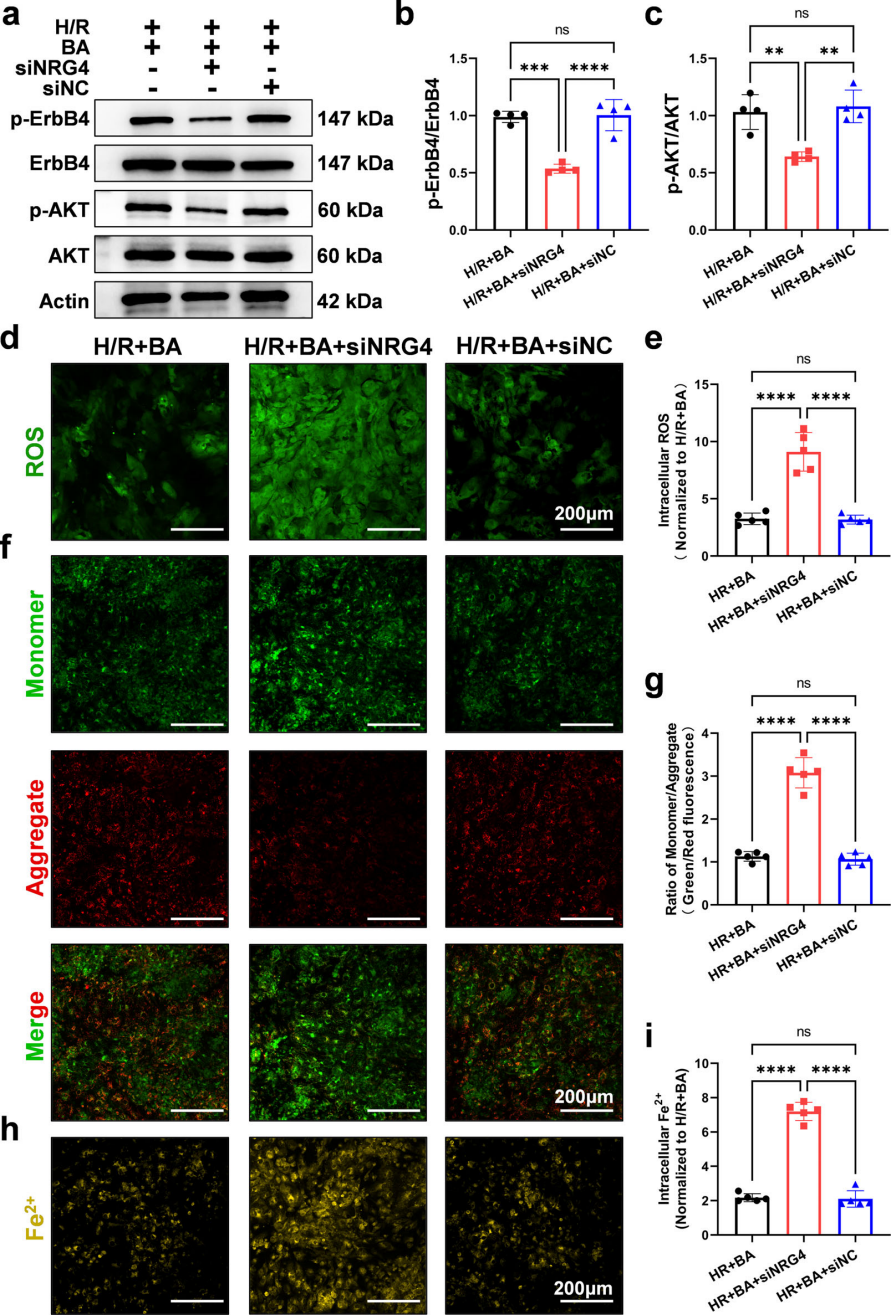
BA sheets activate ErbB4 and suppress ferroptosis in cardiomyocytes in an NRG4–dependent manner following H/R injury. (a) Representative western blots of total and phosphorylated ErbB4 and AKT. (b) Densitometric quantification of total and phosphorylated ErbB4 normalized to actin (n = 4 per group). (c) Densitometric quantification of total and phosphorylated AKT normalized to actin (n = 4 per group). (d) Representative fluorescence images of intracellular ROS in cardiomyocytes. (e) Quantification of fluorescence intensity for ROS (n = 5 per group). (f) JC‐1 staining showing mitochondrial membrane potential (ΔΨm), where red fluorescence (aggregates) indicates polarized mitochondria and green fluorescence (monomers) indicates depolarized mitochondria. (g) Quantification of fluorescence intensity for JC‐1 ratio (monomer/aggregate) (n = 5 per group). (h) Fluorescent images of intracellular ferrous iron (Fe^2+^) levels detected by FerroOrange fluorescence probe. (i) Quantification of fluorescence intensity for intracellular Fe^2+^ (n = 5 per group). The same scale bars (200 µm) were used for all three groups (H/R+BA, H/R+BA+siNRG4, and H/R+BA+siNC). Data are presented as mean ± SD. Statistical significance was determined by one‐way ANOVA followed by Tukey's post‐hoc test. ^**^ *p* < 0.01; ^***^ *p* < 0.001; ^****^ *p* < 0.0001; ns, not significant.

To further verify whether NRG4 mediates the activation of ErbB4 signaling and the anti‐ferroptotic effects of BA sheets, NRG4 was specifically silenced in BA sheets by siRNA transfection. The efficiency of NRG4 knockdown was confirmed by western blot analysis of NRG4 expression and ELISA measurement of NRG4 levels in the conditioned medium. Among the three tested siRNAs, siNRG4‐1 exhibited the highest knockdown efficiency and was therefore selected for subsequent experiments (Figure S6a–c). Cardiomyocytes subjected to H/R were then treated with conditioned media collected from BA sheets (H/R+BA), NRG4‐silenced BA sheets (H/R+BA+siNRG4), and negative control siRNA‐treated BA sheets (H/R+BA+siNC). Western blot analysis of ErbB4 and downstream AKT phosphorylation, combined with ferroptosis‐related assays (ROS, ΔΨm, and intracellular Fe^2^^+^), demonstrated that NRG4 knockdown markedly reduced ErbB4 and AKT phosphorylation and abolished the anti‐ferroptotic effects observed in the H/R+BA group. In contrast, ErbB4 activation, downstream AKT phosphorylation, and ferroptosis suppression were preserved in the H/R+BA+siNC group (Figure 8a–i). These findings indicate that NRG4 derived from BA sheets mediates ErbB4 activation and contributes to the suppression of ferroptosis in cardiomyocytes.

In summary, we constructed BA sheets and demonstrated that they protect cardiomyocytes against H/R‐induced ferroptosis by modulating the NRG4–ErbB4 axis and activating the PI3K/AKT pathway. This in turn promotes NRF2 nuclear translocation and HO‐1 expression, thereby enhancing antioxidant capacity and suppressing ferroptotic cell death.

## 4 Discussion and Conclusion

**FIGURE 9:**
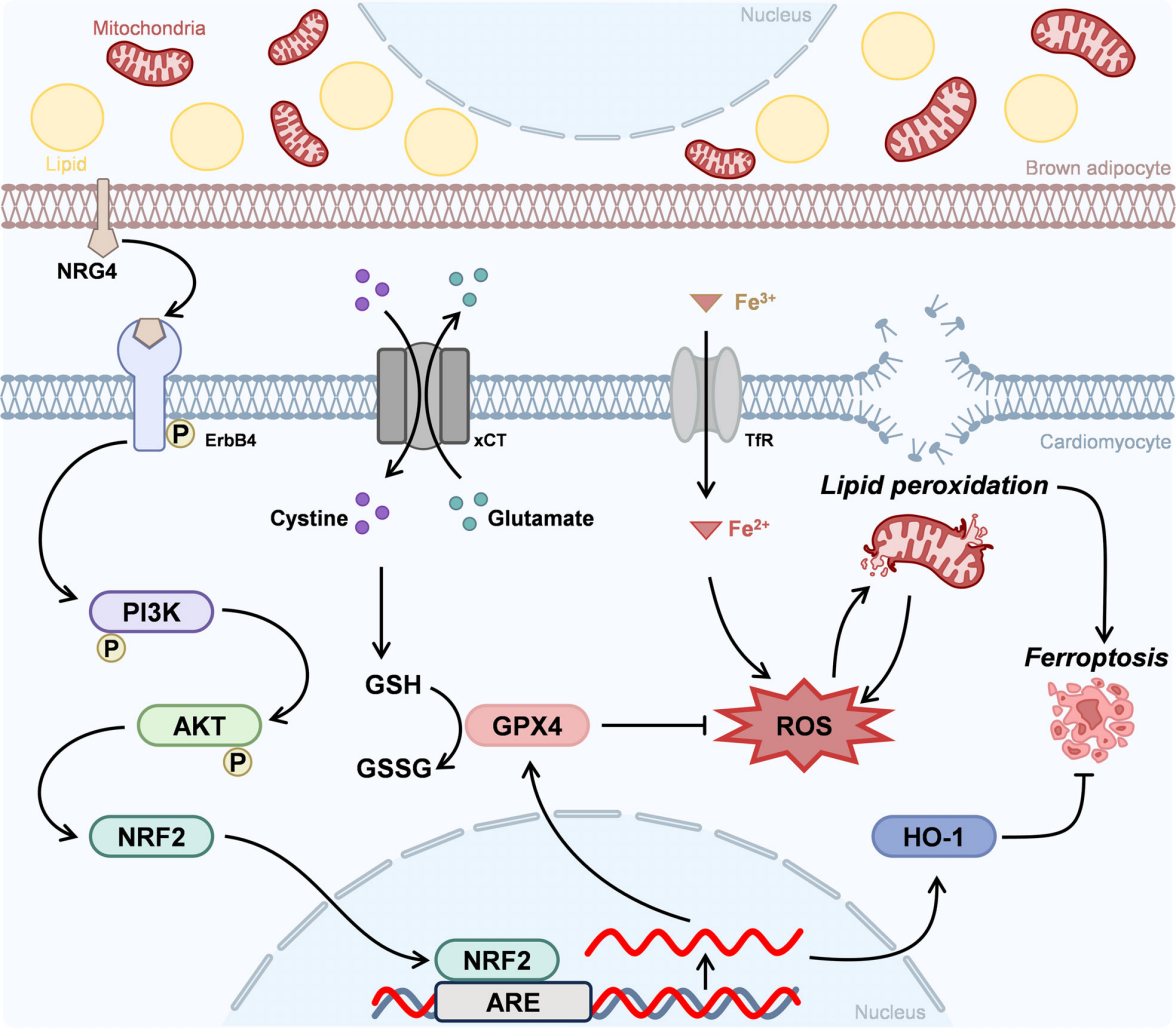
Schematic diagram of the underlying mechanism of BA sheets‐mediated therapy for myocardial I/R injury.

We constructed BA sheets, investigated their cardioprotective effects against myocardial I/R injury, and elucidated their underlying mechanisms. In a rat model, the transplantation of the BA sheets onto the injured heart surface significantly improved cardiac function, reduced ischemic size and fibrosis, alleviated ventricular remodeling, and promoted angiogenesis. In vitro, conditioned medium from the BA sheets enhanced cardiomyocyte survival and contractility, while suppressing apoptosis under H/R conditions. This markedly inhibited ferroptosis, a key form of regulated cell death in I/R injury. Moreover, the blockade of the NRG4–ErbB4 axis abolished these protective effects, underscoring its essential role. Mechanistically, our findings demonstrate for the first time that the BA sheets protect against myocardial I/R injury by activating the PI3K/AKT and NRF2/HO‐1 signaling pathways via NRG4–ErbB4 activation, thereby suppressing ferroptosis (Figure 9).

Myocardial I/R injury is a prolonged, multifaceted pathological process [33]. Various forms of cell death, including apoptosis [34], necrosis [35], autophagy [36], pyroptosis [37], and ferroptosis [38], contribute to its progression, with ferroptosis being predominant in later stages [8]. Although several ferroptosis‐targeting strategies have been proposed in the clinical context, effective interventions remain elusive. For example, glucocorticoids, which are commonly used to manage inflammation, allergies, and shock, hypothetically alleviate PCI‐related coronary inflammation. Despite their theoretical benefits, clinical data do not support the cardioprotective effects of glucocorticoids on myocardial I/R injury [39]. Other studies using isolated heart perfusion and coronary ligation models have demonstrated that iron chelators can reduce myocardial damage by suppressing ferroptosis [40, 41, 42]. However, the clinical use of iron chelators is limited by side effects, including gastrointestinal symptoms, liver damage, arthropathy, neutropenia, agranulocytosis, and zinc deficiency in patients with diabetes [43]. Thus, dissecting the cellular pathways and potential upstream regulators of ferroptosis is essential to identify novel, clinically translatable cardioprotective strategies.

BAT has received renewed attention after its identification in adults approximately 15 years ago [44]. Unlike white adipose tissue, which primarily functions as an energy‐storage depot, BAT specializes in energy expenditure by dissipating chemical energy as heat via nonshivering thermogenesis [45]. This distinctive thermogenic capacity allows BAT to contribute to the systemic energy balance and metabolic regulation [13]. BAT is a source of endocrine factors capable of regulating systemic metabolism, highlighting its potential as a therapeutic target in obesity and metabolic disorders [46]. Recent studies have begun to explore the cardiovascular effects thereof, primarily through the manipulation of the systemic function of BAT, either by surgical excision or ectopic transplantation, such as in the epididymal region [15, 18]. These approaches leverage BAT‐derived secretory factors but do not directly target the ischemic myocardium. However, the local paracrine effects of BAT and BAs on myocardial I/R injury remain unclear.

To the best of our knowledge, this is the first study to engineer BA sheets in vitro and directly transplant them onto the epicardial surface to treat myocardial I/R injury. This strategy was designed to enable BAs to exert localized and efficient therapeutic effects during the early phase of reperfusion. Echocardiographic analysis on day 1 post‐transplantation did not show a significant improvement in cardiac function. However, TTC/Evans Blue staining demonstrated a marked reduction in IS in the I/R+BA group at this early stage, suggesting rapid therapeutic efficacy (Figure 1c,i). This may be because, despite differences in IS on day 1, global cardiac compensatory mechanisms were still preserved and concealed the impact of regional injury on overall function, resulting in no detectable differences in cardiac function from echocardiography at this early time point [47]. After day 1, cardiac function in the I/R group progressively declined, whereas the I/R+BA group exhibited sustained improvement, consistent with the observed anti‐fibrotic and anti‐remodeling effects (Figures 1c,d, and 3b,e). Furthermore, extended observation to 28 days and inclusion of additional control groups confirmed the durability and specificity of BA sheet‐mediated cardioprotection. Neither PreBA nor WA sheets alleviated cardiac function or remodeling, whereas BA sheets maintained stable functional benefits and reduced fibrosis and hypertrophy over time. These findings highlight the sustained and cell‐type‐specific therapeutic effects of BA sheets (Figures S2e,f, and S3). The BA sheets treatment led to the highest vascular density in the peri‐infarct region, whereas no significant differences were detected between the Sham and I/R groups (Figure 2h). However, HUVECs assays showed that H/R impaired endothelial function in vitro, which was effectively restored by BA sheets treatment (Figures S4 and S5). The discrepancy between the in vivo and in vitro findings may be due to the presence of collateral circulation in vivo, which can obscure I/R‐induced angiogenic impairment. In contrast, all cells in vitro were uniformly subjected to H/R stress, revealing a direct proangiogenic effect of the BA sheets. Nevertheless, both models consistently demonstrated the proangiogenic capacity of the BA sheets, reinforcing their potential to promote vascular regeneration after I/R injury.

Having confirmed the cardioprotective effects of the BA sheets in vivo, we sought to explore the underlying mechanisms thereof. Using an in vitro H/R model of cardiomyocytes, we verified that the BA sheets enhanced cell viability and contractile function in vitro. In addition, the treatment attenuated H/R‐induced apoptosis, which is consistent with previous findings [15], suggesting that BA sheets support cardiomyocyte survival under H/R‐induced stress (Figure 3). These observations prompted us to investigate whether BA sheets were involved in the cardioprotective effects of ferroptosis.

BAs secrete various factors, including NRG4, a member of the neuregulin family [46]. NRG4 is expressed in specific peripheral tissues, such as the lungs, liver, and adipose tissue, with the highest expression level observed in BAT [30]. It specifically binds to ErbB4 receptors on target cells, thereby activating downstream pathways including the PI3K/AKT and NRF2/HO‐1 pathways, both of which are implicated in regulating oxidative stress and cell survival [48]. Furthermore, the PI3K/AKT pathway enhances cellular antioxidant capacity by promoting the expression of key antiferroptotic regulators such as GPX4 and xCT, while simultaneously reducing lipid peroxidation [49, 50]. In parallel, the NRF2/HO‐1 axis serves as a master regulator of redox homeostasis. Upon activation, NRF2 translocates into the nucleus and binds to the antioxidant response element in the promoter regions of target genes, inducing the transcription of a suite of antioxidant and iron metabolism‐related genes [51]. Among these genes, HO‐1 plays a crucial role in degrading heme into biliverdin, carbon monoxide, and free iron, thereby limiting labile iron accumulation, which is a key driver of ferroptosis [52].

Based on this knowledge and our RNA sequencing results, we hypothesized that the BA sheets suppress ferroptosis in cardiomyocytes under H/R conditions by secreting NRG4, which, in turn, activates ErbB4 and the downstream PI3K/AKT and NRF2/HO‐1 signaling pathways. To test this hypothesis, H/R+BA cardiomyocytes were treated with an ErbB4 inhibitor. The results showed that the BA sheets significantly suppressed ferroptosis‐related markers under H/R conditions; however, this protective effect was markedly diminished upon inhibition of ErbB4 (Figure 5). Furthermore, the treatment preserved the mitochondrial structure and function, as evidenced by the improved mitochondrial membrane potential and reduced ultrastructural damage observed by TEM (Figure 6). Western blot analysis also confirmed that BA sheets promote the activation of the PI3K/AKT and NRF2/HO‐1 pathways, whereas the inhibition of ErbB4 reverses these molecular changes (Figure 7). To further substantiate the mechanistic role of NRG4, loss‐of‐function experiments were performed using siRNA‐mediated knockdown in BA sheets (Figure S6.) Silencing NRG4 markedly attenuated ErbB4 phosphorylation and abolished the anti‐ferroptotic effects observed in the H/R+BA group, whereas these effects were preserved in the H/R+BA+siNC group (Figure 8). These findings provide direct evidence that NRG4 acts as a key paracrine mediator linking BA sheets to ErbB4 activation and downstream ferroptosis suppression in cardiomyocytes following H/R injury. Collectively, these findings suggest that the NRG4–ErbB4 axis plays a critical role in mediating the antiferroptotic and cardioprotective effects of BA sheets through the activation of PI3K/AKT and NRF2/HO‐1 signaling and the preservation of mitochondrial integrity.

Our findings highlight the translational potential of BA sheets‐based therapies for ischemic heart disease. Given the high incidence and clinical burden of myocardial I/R injury, the development of localized treatments that can be directly applied to the injured regions is of great clinical value. As bioactive cells, BAs can be engineered into transplantable constructs, offering a feasible and localized therapeutic approach. Beyond the heart, such strategies may also be extended to other organs affected by I/R injury, such as the brain, liver, and kidneys, broadening the scope of application of the BA sheets‐based interventions. However, this study had some limitations. Although our research yielded reliable results in a rat I/R injury model, species differences may have affected the broader applicability of the effects of the BA sheets. Additionally, the cardioprotective role of the BA sheets may involve other signaling pathways or secreted factors that have not yet been explored. Future studies should investigate the interactions between different signaling pathways in cardiomyocyte survival and explore the potential effects of BA sheets on other cardiovascular pathological conditions. Furthermore, although this study confirmed the inhibitory effect of BA sheets on ferroptosis, the role of other forms of cell death, such as apoptosis, necrosis, and autophagy, in I/R injury warrants further investigation. Moreover, the limited availability of BAs may limit the broad application of BA sheets. However, the differentiation of readily accessible cell types such as mesenchymal stem cells into BAs offers a potential solution [53].

In conclusion, we successfully constructed BA sheets and demonstrated for the first time that they can alleviate myocardial I/R injury via NRG4–ErbB4 axis–mediated activation of the PI3K/AKT and NRF2/HO‐1 signaling pathways, thereby suppressing ferroptosis. These findings provide a theoretical basis for the therapeutic application of BA sheets in cardiovascular diseases and suggest new avenues for BA sheets‐based treatment strategies.

## Author Contributions

L.S., J.L. and L.L. conceived and designed the study, including the central hypothesis, project framework and experimental strategy. L.S., Y.L. and J.Z. performed the animal experiments under the supervision of T.K. and S.M. L.S. and Y.L. conducted cell culture and molecular biology experiments. L.S., Y.L. and J.L. analyzed and interpreted the experimental data. L.S. performed data visualization, prepared the figures, and drafted the initial version of the manuscript. J.L., L.L. and S.M. acquired funding, administered the project, and supervised the overall study. All authors contributed to data interpretation, critically reviewed the manuscript, and approved the final version for submission.

## Conflicts of Interest

The authors declare that they have no conflict of interest.

## Supporting information




**Supporting File 1**: advs75588‐sup‐0001‐SuppMat.pdf.

**Supporting File 2**: advs75588‐sup‐0002‐VideoS1.avi.

**Supporting File 3**: advs75588‐sup‐0003‐VideoS2.avi.

**Supporting File 4**: advs75588‐sup‐0004‐VideoS3.avi.

## Data Availability

The data that support the findings of this study are available from the corresponding author upon reasonable request.
