# Supplementary material for: Brown Adipocyte Sheets Alleviate Myocardial Ischemia–Reperfusion Injury Through NRG4–ErbB4–Dependent Ferroptosis Inhibition
Source: Adv Sci (Weinh). 2026 May 28;13(43):e75588. doi: 10.1002/advs.75588 (PMC13336044; doi:10.1002/advs.75588)
Supplement: Supplementary file 1 — Supporting File 1: advs75588‐sup‐0001‐SuppMat.pdf. [file ADVS-13-e75588-s001.pdf]

Supporting Information

Brown Adipocyte Sheets Alleviate Myocardial Ischemia-Reperfusion Injury Through NRG4–ErbB4–Dependent Ferroptosis Inhibition

Lifu Sun *et al.*

*Corresponding authors: Junjun Li Email: lijunjun@ap.eng.oasak-u.ac.jp
Li Liu Email: liuli@ap.eng.osaka-u.ac.jp
Shigeru Miyagawa Email: miya-p@surg1.med.osaka-u.ac.jp

This file includes:

Supplementary Figure S1 to S6
Supplementary Table S1

Other Supplementary Materials for this manuscript include the following:

Videos S1 to S3

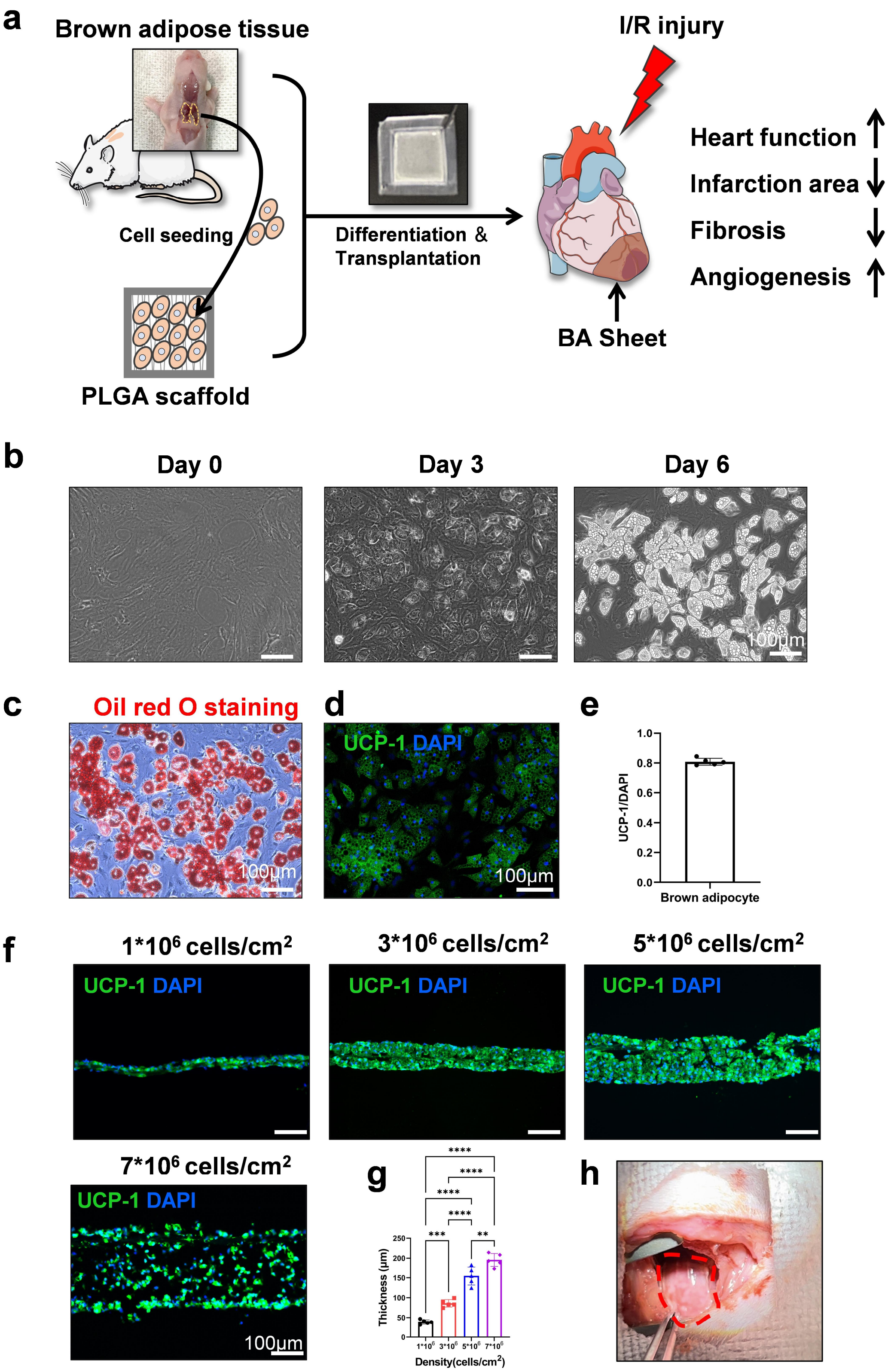

Supplementary Figure S1. Characterization of BAs and BA sheets.

a, Schematic diagram showing the establishment of BA sheets and subsequent transplantation procedures. **b,** Representative brightfield images of BAs on day 0, 3, and 6 of *in vitro* differentiation. **c,** Oil Red O staining demonstrating lipid droplet accumulation in the BAs on day 6. **d,** Immunofluorescence staining

of UCP-1 (green) in differentiated BA nuclei were counterstained with DAPI (blue). **e,** Quantification of UCP-1 fluorescence intensity normalized to DAPI (n = 5). **f,** Representative cross-sectional cryosection images of BA sheets constructed at varying seeding densities (1×10, 3×10, 5×10, and 7×10 cells/cm^2). **g,** Quantification of sheets thickness corresponding to different cell densities (n = 5 per group). **h,** Image of a BA sheet transplanted onto the damaged myocardium, highlighted by the red dashed outline. Scale bars: 100 µm. Data are presented as mean ± SD. Statistical significance was determined using one-way analysis of variance (ANOVA), followed by Tukey's post-hoc test. $^{**}p < 0.01$; $^{***}p < 0.001$; $^{****}p < 0.0001$.

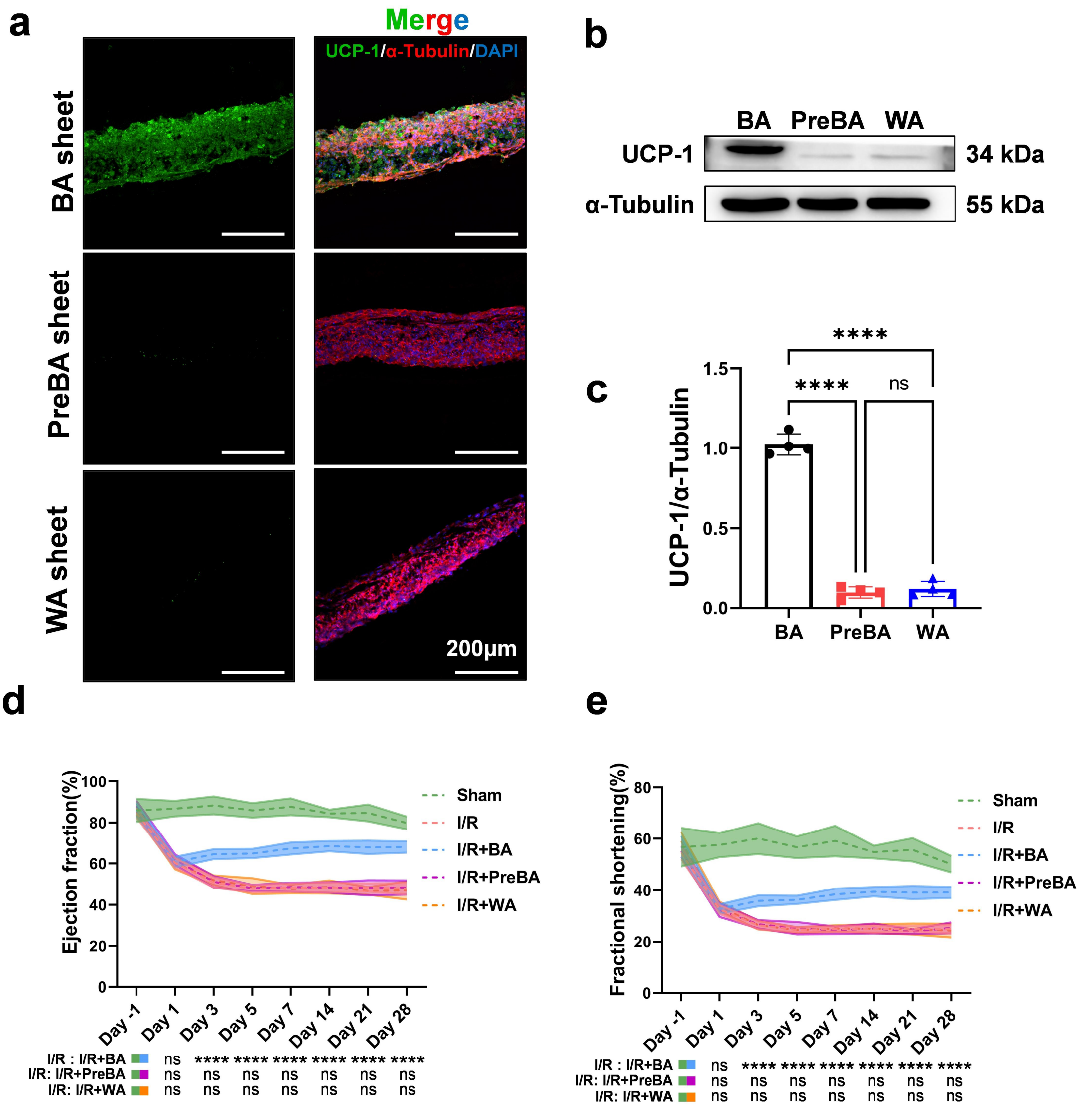

Supplementary Figure S2. Characterization of different sheets and their long-term effects on cardiac function after I/R injury.

a, Immunofluorescence staining of BA, PreBA, and WA sheets for UCP-1 (green, brown adipocyte marker), α-tubulin (red), and DAPI (blue). **b,** Western blot analysis of UCP-1 and α-tubulin expression in BA, PreBA, and WA sheets. **c,** Densitometric quantification of UCP-1 expression normalized to the BA group (n = 4 per group). **d, e,** Cardiac function assessed by echocardiography, represented as LVEF (d) and LVFS (e), at baseline (day −1) and days 1, 3, 5, 7, 14, 21, and 28 after I/R injury (Sham: n = 5; I/R: n = 6; I/R+BA: n = 6; I/R+PreBA: n = 5; I/R+WA: n = 5). Data are presented as mean ± SD. Statistical significance was determined by one-way ANOVA followed by Tukey's post hoc test. ****$p < 0.0001$; ns, not significant.

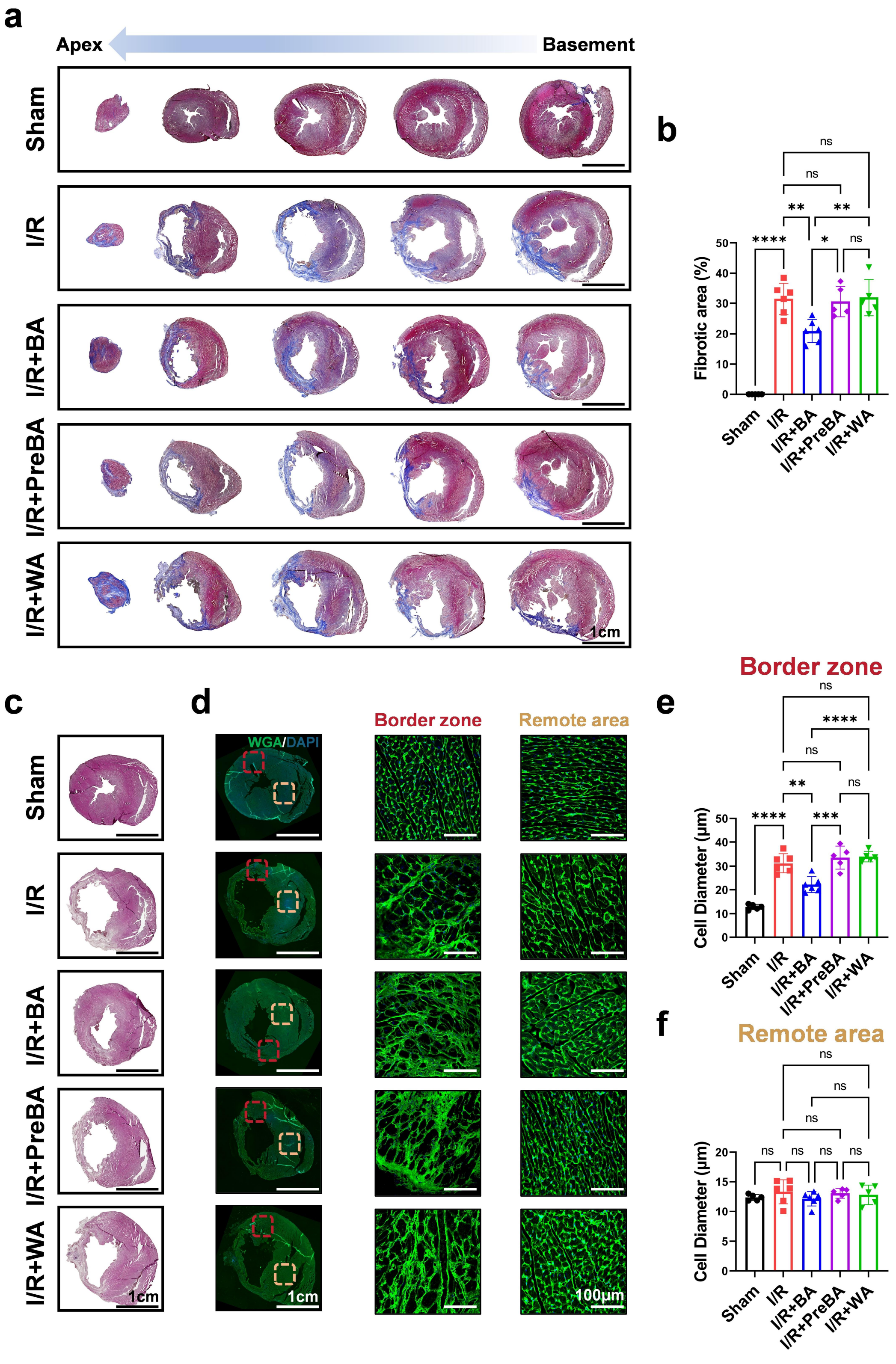

Supplementary Figure S3. Long-term effects of different adipocyte sheets on cardiac fibrosis and ventricular remodeling at 28 days after I/R injury.

a, Masson's trichrome staining of heart sections 28 days postreperfusion. The fibrotic areas are stained blue (scale bar = 1 cm). **b,** Quantification of fibrotic area as a percentage of the total myocardial area. **c,**

HE staining of heart sections (scale bar = 1 cm). **d,** WGA staining of heart sections, highlighting cardiomyocyte membranes (green). Images are shown for each group, including original sections and magnified views of both the border (red box) and remote zones (yellow box). Original image scale bar = 1 cm; magnified view scale bar = 100 μm. **e-f,** Quantitative analysis of cardiomyocyte diameter in the border zone (e) and remote zone (f). The same group sizes (Sham: n = 5; I/R: n = 6; I/R+BA: n = 6; I/R+PreBA: n = 5; I/R+WA: n = 5) were used for all quantifications. Data are presented as mean ± SD. Statistical significance was determined using a one-way ANOVA followed by Tukey's post-hoc test. **p* < 0.05; ***p* < 0.01; ****p* < 0.001; *****p* < 0.0001; ns, not significant.

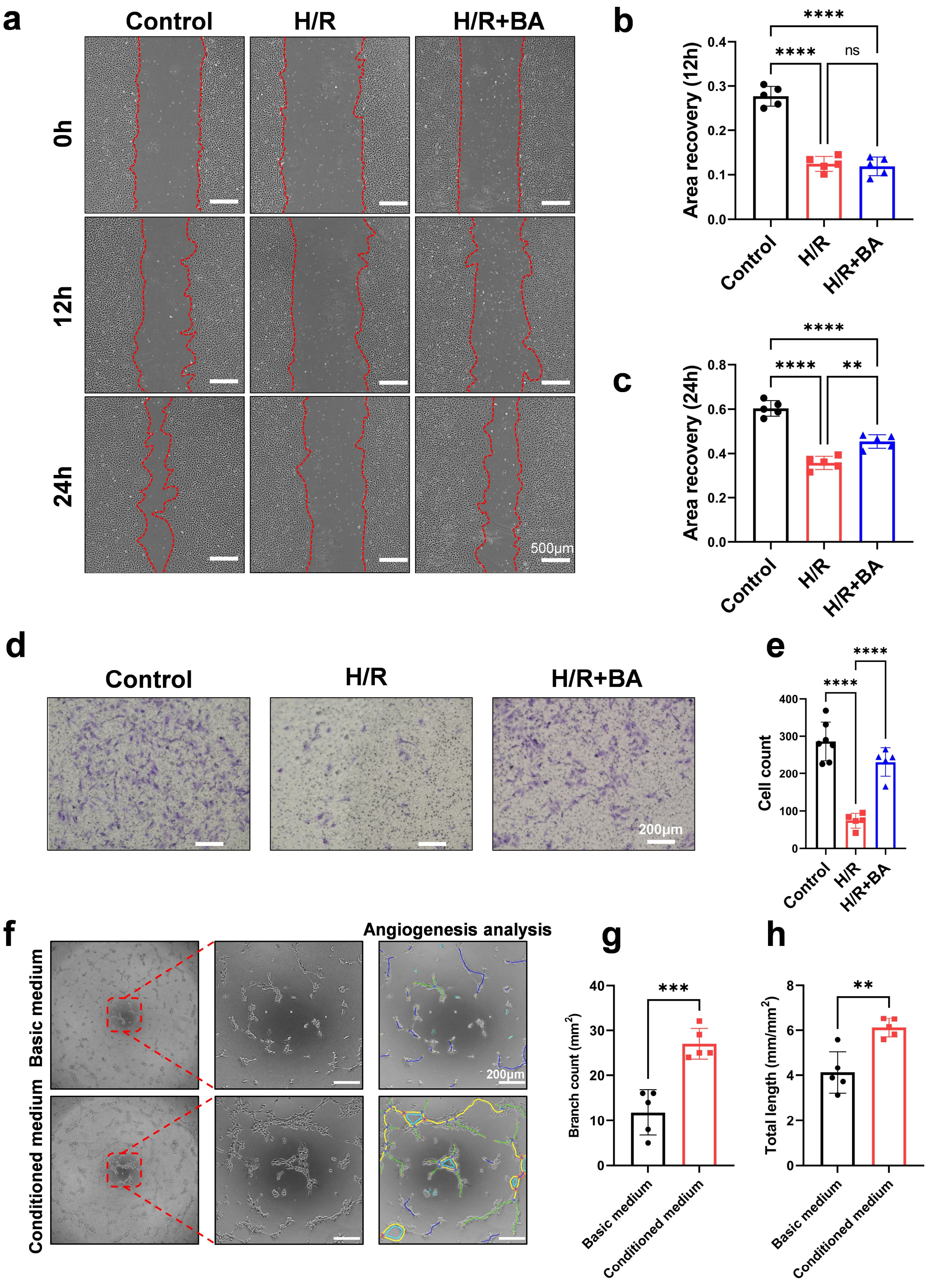

Supplementary Figure S4. BA sheets promote angiogenic potential and migration of endothelial cells under H/R conditions.

a, Representative bright-field images of scratch wound healing assays in HUVECs under control, H/R, and H/R + BA conditions at 0 h, 12 h, and 24 h. Red lines indicate the wound edges. Scale bars: 500 μm. **b,** Quantification of wound closure area at 12 h (n = 5 per group). **c,** Quantification of wound closure

area at 24 h (n = 5 per group). **d,** Representative bright-field images of Transwell migration assays in control, H/R, and H/R + BA groups. Scale bars: 200 μm. **e,** Quantification of migrated cell number per field (n = 5 per group). **f,** Representative images of *in vitro* tube formation assay using basic medium and BAs-conditioned medium. Images were taken at 4 h and include magnified views of the central area to show capillary-like structures. Scale bars: 200 μm. **g,** Quantification of branch points (n/mm^2) (n = 5 per group). **h,** Quantification of total tube length (mm/mm^2) (n = 5 per group). Data are presented as mean ± SD. Statistical significance was determined using one-way ANOVA followed by Tukey's post hoc test. $^{**}p < 0.01$; $^{***}p < 0.001$; $^{****}p < 0.0001$; ns, not significant.

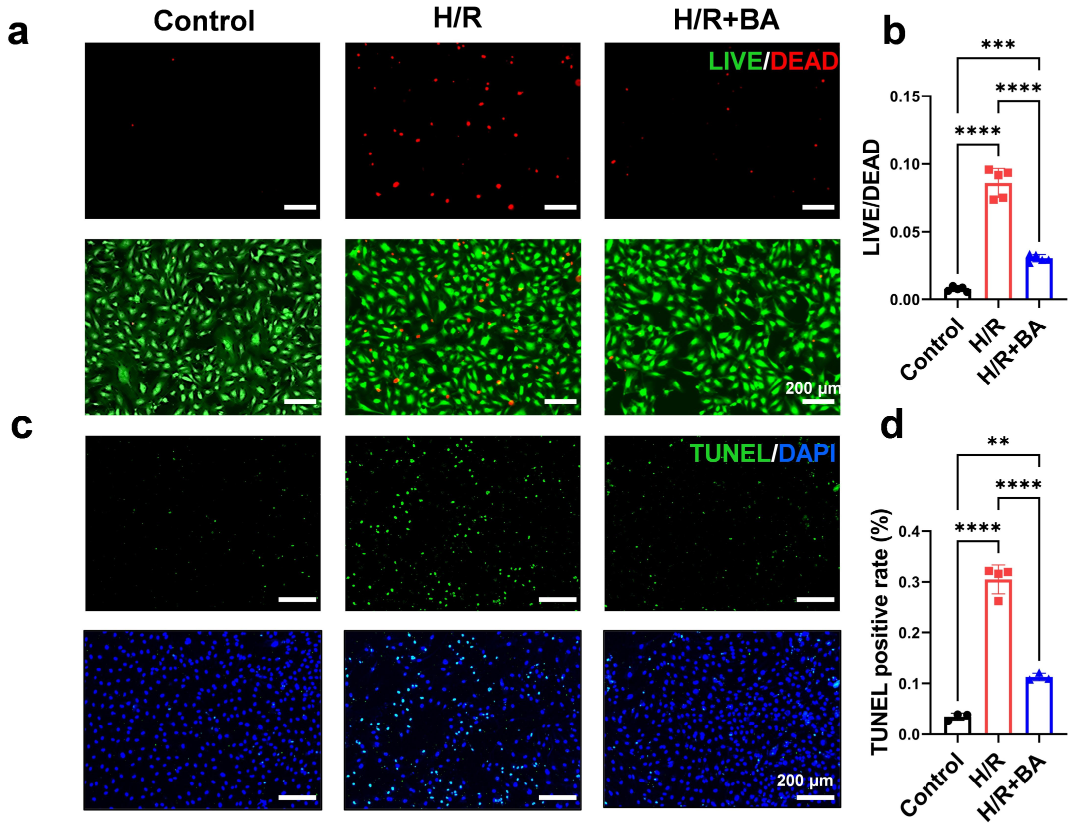

Supplementary Figure S5. BA sheets protect endothelial cells from cell death and apoptosis under H/R conditions.

a, Representative fluorescence images of Live/Dead staining in HUVECs under Control, H/R, and H/R + BA conditions. Live cells are labeled in green; dead cells are labeled in red. Scale bars: 200 μm. **b,** Quantification of the percentage of live/dead in each group (n = 5 per group). **c,** Representative TUNEL staining images showing apoptotic nuclei (green) and total nuclei (DAPI, blue). Apoptotic cells are indicated by green and blue co-localization. Scale bars: 200 μm. **d,** Quantification of TUNEL-positive nuclei normalized to DAPI (Control, n = 3; H/R, n = 4; H/R+BA, n = 3). Data are presented as mean ± SD. Statistical significance was determined using one-way ANOVA followed by Tukey's post hoc test. $^{**}p < 0.01$; $^{***}p < 0.001$; $^{****}p < 0.0001$.

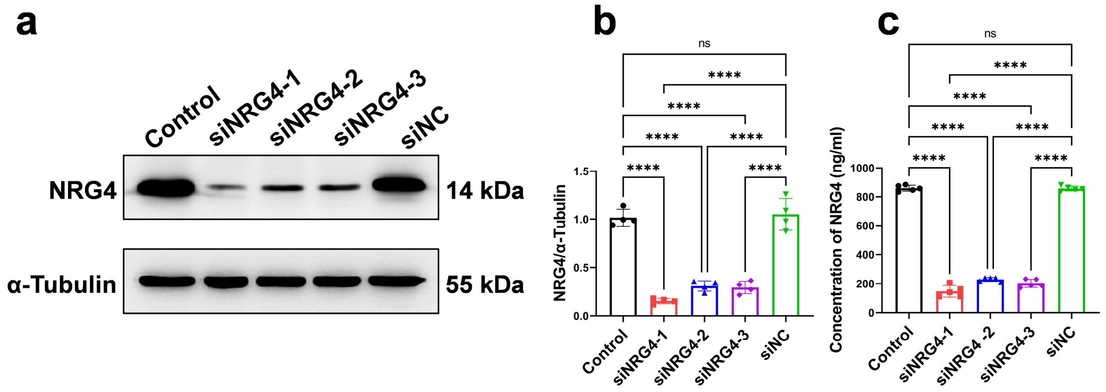

Supplementary Figure S6. Validation of NRG4 knockdown efficiency in BA sheets.

a, Representative western blot of NRG4 in BA sheets. **b,** Densitometric quantification of NRG4 expression normalized to the control group (n = 4 per group). **c,** NRG4 concentrations in conditioned media from BA sheets in each group (n = 5 per group). Data are presented as mean ± SD. Statistical significance was determined by one-way ANOVA followed by Tukey's post hoc test. ****$p < 0.0001$; ns, not significant.

Supplementary Table S1. Sequences of siRNAs used in this study

Name	Target gene	Sense (5′–3′)	Antisense (5′–3′)
**siNRG4-1**	**rat NRG4**	GCAGAAUUAUGUUUAGUGAGCdTdT	UCACUAAACAUAAUUCUGCUGdTdT
**siNRG4-2**	**rat NRG4**	GGUGCUGUUUCAACCCUAAUCdTdT	UUAGGGUUGAAACAGCACCUCdTdT
**siNRG4-3**	**rat NRG4**	CAGAGCUACUGGAAUUAAAGGdTdT	UUUAAUUCCAGUAGCUCUGAGdTdT
**siNC**	–	UUCUCCGAACGUGUCACGUdTdT	ACGUGACACGUUCGGAGAAdTdT

Supplementary Videos S1–S3. Bright-field videos showing the beating activity of cardiomyocytes under different conditions.

Video **S1**, Control group; Video **S2**, H/R group; Video **S3**, H/R + BA group. Scale bars: 100 μm.
